# Dermal Permeability of Perfluorohexane Sulfonic Acid (PFHxS) and Perfluorohexanoic Acid (PFHxA) in Varying Vehicles and the Effects on Skin Integrity in a Reconstructed Human Epidermis Model

**DOI:** 10.3390/toxics14090756

**Published:** 2026-08-26

**Authors:** Lisa M. Weatherly, Notashia N. Baughman, Callee M. Walsh, Laurel G. Jackson, Ewa Lukomska, Madison P. Cooper, Jason E. Ham, Stacey E. Anderson

**Affiliations:** 1 Allergy and Clinical Immunology Branch, Health Effects Laboratory Division, National Institute of Occupational Safety and Health, Morgantown, WV 26505, USA; 2 Chemical and Biomonitoring Branch, Health Effects Laboratory Division, National Institute of Occupational Safety and Health, Morgantown, WV 26505, USA

**Keywords:** PFAS, dermal absorption, dermal penetration, PFHxS, PFHxA, reconstructed human epidermis, skin barrier integrity

## Abstract

Per- and polyfluoroalkyl substances (PFAS) are widespread environmental contaminants associated with adverse health effects, yet dermal exposure remains poorly characterized. This study evaluated the influence of vehicle and chemical structure on dermal absorption and skin barrier integrity using a reconstructed human epidermis model (EpiDermFT). Two C6 PFAS, perfluorohexane sulfonate (PFHxS) and perfluorohexanoic acid (PFHxA), were applied in three vehicles (acetone, water, and diethylene glycol monobutyl ether [DEGME]) at concentrations of 0–0.015% for 4 or 24 h. PFAS concentrations were quantified in tissue and receptor media, and skin barrier integrity was assessed using histological and immunological endpoints. Increasing exposure resulted in higher concentrations of PFHxS and PFHxA in both tissue and media across all vehicles, indicating dermal penetration and absorption. PFHxS exhibited greater tissue retention but only a few small, isolated changes in cytokine and gene expression and no significant effect on barrier integrity. In contrast, PFHxA induced increased expression of inflammatory mediators and alterations in skin barrier function. These findings indicate a divergence between dermal retention and biological activity. Overall, while the vehicle influenced PFAS transport, biological responses were more strongly associated with functional group. These results suggest that functional group-dependent toxicity is an important determinant of dermal PFAS effects and should be considered when characterizing PFAS dermal hazard and grouping structurally related compounds for assessment.

## 1. Introduction

Per- and polyfluoroalkyl substances (PFAS) are a large, diverse class of synthetic chemicals that are characterized by multiple carbon–fluorine (C–F) bonds. These bonds contribute to PFAS thermal and chemical stability, persistence, and oil- and water- repellency [1,2,3,4]. These properties have contributed to the incorporation of PFAS in a variety of products such as for stain-resistance and waterproofing in textiles, clothing, and carpets, grease-resistant food packaging, electronics, medical devices, personal-care products and cosmetics, and firefighting foams [3,5,6,7]. These products put human skin in routine contact with PFAS, leading to the potential for dermal exposure via commercial products, the environment, and occupationally.

Despite the potential for dermal exposure, very few studies address this exposure route and the influence of vehicle or substrate. However, studies have found PFOA to penetrate skin [8,9]. Historically, many studies have characterized dermal PFAS uptake as limited compared with ingestion or inhalation [3]. Emerging data, however, suggest that the dermal pathway may be more important than once assumed, especially for certain PFAS-containing products [10,11]. Recent in vitro human-skin models reported measurable skin penetration for many PFAS, with some short-chain compounds showing higher permeability than legacy long-chain molecules [10,12]. In cosmetics and personal-care products, where there is the most prolonged direct skin contact, modeling indicates that dermal intake of short-chain PFAS can rival or exceed dietary intake in frequent users [12]. Additionally, several occupational groups are exposed to PFAS, including firefighters and support services, ski wax applications, and manufacturing plants [13,14,15,16]. Firefighters are exposed via the dermal route by using aqueous film-forming foams (AFFFs) and the use of PFAS-coated personal protective equipment [17,18,19,20]. Several studies show that firefighters consistently have higher levels of PFAS in their serum compared to the other groups [15,21,22].

Perfluorohexane sulfonic acid (PFHxS) and perfluorohexanoic acid (PFHxA) are both six-carbon (C-6) PFAS but differ in their functional group (sulfonate vs carboxylate, respectively). PFHxS has a long half-life of 4.7–35 years in humans [3], while PFHxA has a much shorter half-life of 32 days [23]. Even though PFHxS production was discontinued in the USA in the early 2000s, recent studies are still showing detectable levels in both human samples and drinking water [24,25]. Human serum PFHxS is detected in ~96–99% of the general population at geometric means of roughly 1–3 ng/mL [26,27], rising to ~4–6 ng/mL in occupationally exposed firefighters [15,20], whereas PFHxA is infrequently detected in serum (often <0.5% of samples) owing to its short half-life and is more frequently detected in urine [26,28]. Epidemiological studies suggest an association between PFHxS exposure and increases in serum hepatic enzymes (alanine aminotransferase) and decreases in serum bilirubin levels [3]. Individual epidemiological studies have also observed associations between PFHxS (~3.0 ng/mL) and certain types of cancers in humans [29,30]. However, the EPA IRIS Toxicological Review of PFHxS Report recently concluded that there is inadequate information to assess the carcinogenic potential of PFHxS [31]. In animal studies, the liver was found to be a sensitive target for PFHxS exposure along with developmental endpoints [3]. Additional animal studies show effects on bone health at 2 mg/L [32], immune function at 30 µM [33], and sperm function at 0.1 mg/kg/day [34]. PFHxA has less toxicological data available, but individual studies have shown an association with exposure in heart disease (0.018 ng/mL in plasma) [35] and cancers [29] in humans. A U.S EPA review of PFHxA published in 2023 concluded that there is inadequate information to assess the carcinogenic potential of PFHxA [36]; however, they did find that the available evidence indicates PFHxA exposure is likely to cause negative endocrine, hepatic, developmental, and hematopoietic effects in humans, given sufficient exposure conditions.

Our lab has previously shown that both PFHxS and PFHxA (1.25–5%) are absorbed through mouse skin after dermal exposure, resulting in systemic toxicological effects [37,38]. To investigate this further and translate these findings to a more realistic occupational situation, a human skin equivalent model was utilized for the studies described in this paper. Reconstructed human epidermis (RHE) models have successfully been used to evaluate the skin barrier, absorption, penetration, and mechanisms of skin disease [39,40,41]. The Mattek EpiDerm Full Thickness (EpiDermFT) model is a human skin equivalent model composed of human epidermal keratinocytes, consisting of organized and proliferative basal cells, spinous and granular layers, and cornified epidermal layers that are metabolically active [42,43]. This model is cultured at the air–liquid interface (ALI), allowing for investigation of topically applied chemicals. The movement of chemicals through the skin occurs in a two-step sequence. Initially, a substance applied to the skin surface must pass into the outermost protective layer, the stratum corneum. When a molecule reaches this layer, it has undergone dermal penetration, which refers to entry into the skin but not yet through its full thickness. Only after a compound travels beyond the epidermis and enters systemic circulation (in vivo) or media in skin models (in vitro) can it be considered to have been absorbed, indicating the fraction that has successfully crossed the entire skin layer. As the vehicle can influence the compound’s permeability, three vehicles were used to represent relevant PFAS exposure scenarios; water, to mimic environmental exposures; acetone, to serve as a historical control; and DEGME. Acetone was included as a historical laboratory reference vehicle to permit comparison with our prior in vivo murine studies and with earlier work on PFOA skin penetration, rather than as a surrogate for human exposure. Because acetone can alter the ionization state of PFAS and thereby enhance penetration, it also serves as a high-permeability benchmark against which the human-relevant vehicles, water and DEGME, can be compared. DEGME (diethylene glycol monobutyl ether) is an amphiphilic solvent widely used in cosmetic, personal-care, and pharmaceutical formulations, and it is also incorporated into some AFFF formulations to modulate surfactant behavior [44,45], making it a directly exposure-relevant vehicle.

This study aimed to (i) quantify PFAS absorption under different exposure conditions, (ii) compare biological responses associated with functional group differences, and (iii) determine the relative importance of vehicles versus chemical structure in driving dermal toxicity by using structurally distinct C6 PFAS, PFHxA and PFHxS, in a reconstructed human epidermis model. These compounds were selected to isolate the influence of the functional group (carboxylate vs sulfonate) while maintaining the same carbon chain length. In addition, three representative vehicles were used to evaluate the role of solvent conditions relevant to environment, laboratory, and consumer product exposures. The findings provide insight into structure–activity relationships for PFAS and have implications for improving dermal exposure assessment and risk evaluation in environmental and occupational contexts.

## 2. Materials and Methods

### 2.1. Reconstructed Human Epidermis Model

EpiDermFT (MatTek, Ashland, MA, USA) tissues were equilibrated as per manufacturer instructions. Tissues were placed at 4 °C for 2 h upon arrival, then transferred to a 6-well plate containing 2.5 mL/well of pre-warmed Dulbecco’s Modified Eagle’s Medium (DMEM) containing epidermal growth factor, insulin, hydrocortisone, gentamicin (5 µg/mL), amphotericin B (0.25 µg/mL), phenol red, and other MatTek proprietary stimulators of epidermal differentiation and lipid precursors. Tissues were incubated overnight at 37 °C with 5% CO_2_ to equilibrate. Tissues were washed with 100 µL pre-warmed Dulbecco’s phosphate-buffered saline (DPBS) and the medium was replaced with 2.5 mL hydrocortisone-free, phenol red-free DMEM.

### 2.2. Chemicals and Materials

Acetone [CAS #67-64-1] was purchased from Sigma-Aldrich (St. Louis, MO, USA), HyPure cell culture water was purchased from Cytiva (Marlborough, MA, USA), and diethylene glycol monobutyl ether (DEGME) [CAS #112-34-5] was purchased from TCI American (Portland, OR, USA) through Fisher Scientific. Perfluorohexane sulfonic acid (95%, PFHxS) [CAS #355-46-4], perfluorohexanoic acid (98%, PFHxA) [CAS #307-24-4], and perfluoroheptanesulfonic acid (≤100%, PFHpS) [CAS #375-92-8] were purchased from Synquest Laboratories (Alachua, FL, USA). Perfluoropentanoic acid (PFPeA, 97%), Perfluorooctanesulfonic acid solution (PFOS, 100 ug/mL in methanol, analytical standard), and ammonium acetate (NH_4_OAc, ≥99.0%) were purchased from Sigma-Aldrich. Ammonium hydroxide (NH_4_OH, 28.0–30.0 *w*/*w*%), water (LC-MS grade), methanol (≥99.9%), and acetonitrile (ACN, ≥99.9%) were purchased from Fisher Scientific (Hanover Park, IL, USA). Solutions of PFPeA, PFHpS, and PFOS were prepared in 50% ACN in H_2_O (*v*/*v*) such that the resulting concentration of each was 800 ng/mL, and these were used as internal standards (IS). PFPeA and PFHpS were used as internal standards for the media sample analysis of PFHxA and PFHxS, respectively, and PFOS was used as the internal standard for the tissue sample analysis of PFHxS. In the preparation of all samples and solutions containing PFAS compound(s), polypropylene tubes and LC vials were utilized to avoid losses of PFAS through adsorption to glass.

### 2.3. PFAS Exposures

PFHxS and PFHxA were dissolved in each of the three vehicles (acetone, water, DEGME). DEGME was diluted to 5% in water prior to use to mimic concentrations found in newer AFFF formulations [46]. PFHxS and PFHxA were then dissolved in this vehicle and 100 µL was applied per tissue. EpiDermFT tissues (*n* = 3/group) were exposed on the apical side to 100 µL of acetone, water, or DEGME (vehicles), PFHxS (0–0.015%), or PFHxA (0–0.015%) for 4 or 24 h. Tissues were incubated at 37 °C in 5% CO_2_ during the exposure. Tissues were washed after exposure and used for downstream analysis as described below. The highest concentration tested, 0.015% (*w*/*v*), corresponds to approximately 0.15 mg/mL, or ~15 µg of PFAS applied per tissue in the 100 µL dose. PFAS concentrations were selected based on an initial range finding study with a high concentration that did not induce overt toxicity (plus two serial dilutions).

### 2.4. Preparation of Calibration Standards and Quality-Control Standards

For the cell media sample analysis, standard stock solutions of each compound (PFHxA, PFHxS, and PFHpS) were prepared separately with a concentration of 10 µg/mL in 50% ACN in H_2_O. The calibration standards for PFHxA were prepared by diluting the associated stock solution with 50% ACN in H_2_O, yielding solutions ranging from 20 to 800 ng/mL. A set volume of the PFPeA stock solution was added to achieve 100 ng/mL in each working calibration solution, with a final volume of 500 µL. The calibration standards for PFHxS were prepared the same way, using the PFHxS and PFHpS (IS) stock solutions. Quality-control samples were prepared by spiking Dulbecco’s Modified Eagle Medium (DMEM) with the appropriate volume of PFHxA or PFHxS solution to achieve 100 ng/mL (low) and 500 ng/mL (medium) concentrations. Matrix blanks were also prepared using only DMEM.

The stock and working solutions for tissue sample experiments were prepared in the same fashion as outlined for the media experiments, with the exception that a 10-ng/mL PFOS solution was used in place of PFHpS as the internal standard for PFHxS. Quality-control samples were prepared using naïve tissue samples homogenized as described below. The concentrations of the QC samples were 100 ng/mL and 500 ng/mL analyte. Matrix blanks were prepared using only naïve tissue samples.

### 2.5. PFAS Quantification in Cell Media Samples

The protocol used was modified from that reported by Heo et al. [47]. The basal cell media samples were stored at −80 °C until analysis and were thawed immediately prior to use. These samples comprised the culture media of the simulated epidermis treated with the analyte working solutions prepared in one of three different solvent vehicles: water, acetone, and DEGME. To ensure their concentrations fell within the calibration curve, each media sample, excluding the 0% PFAS controls, was diluted using 50% ACN in H_2_O by the following: for 0.00375%, dilution factor = 2; 0.0075%, dilution factor =5; 0.015%, dilution factor = 10. To a clean 2-mL polypropylene centrifuge tube, 640 µL of 50% ACN in deionized water with 0.2% formic acid (≥99.0%), 70 µL of deionized water (18 MΩ·cm), 10 µL of an 800 ng/mL internal standard solution prepared in 50% ACN in deionized H_2_O (designated above), and 80 µL of the cell media solution were added. This solution was vortexed to ensure thorough mixing and then centrifuged for 4 min at 20,800× *g*. A 700 µL aliquot of the resulting supernatant was loaded onto a Waters (Milford, MA, USA) Oasis WAX 3 mL solid-phase extraction (SPE) cartridge (60 mg, 30 µm), conditioned using 1 mL methanol and 1 mL DI H_2_O. Interferents were removed by washing the cartridge first with 1 mL of 25 mM NH_4_OAc and then with 1 mL of methanol. The analyte was eluted using 2–500 µL aliquots of 5% NH_4_OH into a collection vessel, and the solvent was removed with a vacuum evaporator. The sample was reconstituted in 80 µL of 50% ACN in DI H_2_O, and the solution was transferred to a Costar 0.22 µm cellulose acetate centrifuge tube filter (Corning, Salt Lake City, UT, USA) to remove any particulate matter. The filtered solution was transferred to polypropylene LC vials (Fisher Scientific, Hanover Park, IL, USA) for analysis.

### 2.6. PFAS Quantitation in Tissue Samples

The method used to purify the EpidermFT tissue samples was adapted from Ragnarsdottir et al. [11]. A section of tissue was collected from each tissue sample, weighed, and added to 150 µL of distilled deionized water with a steel bead. Each tissue sample was homogenized on a TissueLyser II (QIAGEN, Germantown, MD, USA) for 3 min at 27 Hz (4 cycles). Samples were centrifuged down and the volume measured. The tissues were then stored at −80 °C until analysis. The tissue samples were thawed immediately prior to use. These samples consist of 0% PFAS controls and 0.015% PFAS treatments in each of the three solvent vehicles (water, acetone, and DEGME). To ensure the samples fell within the calibration curve range, a dilution of 100:1 was applied. Two milliliters of 1% NH_4_OH solution in methanol and 10 µL of 800 ng/mL IS solution were added to the tube containing the homogenized tissue. The resulting mixture was vortexed briefly and shaken for 10 min at room temperature. Following this, it was sonicated for 5 min at 25 °C and subsequently centrifuged for 5 min at 1330× *g*. The supernatant was removed to a new polypropylene tube. The full agitation and extraction procedure was repeated twice more, each time beginning by adding 1 mL of 1% NH_4_OH to the remaining pellet. The supernatant fractions were combined, and the resulting solution was reduced to a volume of approximately 0.5 mL in a warm water bath (~40 °C) under a stream of nitrogen gas. The concentrated sample was loaded onto a Waters Oasis GCB 50 mg/WAX 200 mg SPE cartridges (Fisher Scientific, Hanover Park, IL, USA) (60 µm particle size, 6 cc) that was preconditioned with 2 mL of MeOH and 2 mL of H_2_O. The cartridge was dried under vacuum for 5 min. The analyte was then eluted using 2–750 µL 1% NH_4_OH in methanol into a polypropylene collection vessel. The solvent was removed using nitrogen gas. The resulting residue was reconstituted in 50% ACN in H_2_O (*v*/*v*) for LC-MS analysis.

### 2.7. LC-MS Parameters

A Thermo Scientific LTQ-XL ion trap mass spectrometer coupled with a Vanquish UHPLC system (Thermo Electron North America LLC, Madison, WI, USA) and an InfinityLab Poroshell 120 PFP column (1.9 µm, 3.0 × 100 mm, Agilent Technologies, Santa Clara, CA, USA) equipped with a InfinityLab Poroshell 120 PFP Fast Guard column (3.0 mm, 1.9 µm, Agilent Technologies, Santa Clara, CA, USA) were used for chromatographic separation and mass spectrometric detection. The Vanquish UHPLC was retrofitted with a PFAS analysis kit to minimize background signal (Thermo Electron North America LLC, Madison, WI, USA). Mobile phase A consisted of 99.9% LC-MS grade H_2_O with 0.1% formic acid (*v*/*v*), while mobile phase B comprised 95% MeOH:5% H_2_O (LC-MS grade) with 0.1% formic acid (*v*/*v*). The flow rate throughout the gradient was 0.4 mL/min. The column compartment and autosampler were maintained at 30 °C and 25 °C, respectively. The gradient program was as follows: 0 to 4.0 min., 10% B; 1.0 to 8.0 min., 10–90% B; 8.0 to 11.0 min., 90% B; 11.0 to 11.5 min., 90–10% B; and 11.5 to 19.0 min., 10% B. The ESI source was operated in negative ion mode, with parameters set as follows: ESI source temperature: 200 °C; sheath gas (N_2_) flow rate: 8; auxiliary gas flow rate: 5; capillary voltage: −25 V; capillary temperature: 275 °C; sweep gas rate: 0; spray voltage: 4.00 kV; tube lens: −123.69 V. Spectra were collected in full scan mode with a scan range of *m*/*z* 50.00–550.00. Data collected from these analyses were processed using TraceFinder™ (v.5.1, Thermo Fisher Scientific, Inc., Waltham, MA, USA) using the following extracted ions: PFPeA (*m*/*z*) = 262.90; PFHxA (*m*/*z*) = 312.90; PFHxS (*m*/*z*) = 398.90; PFHpS (*m*/*z*) = 448.90; PFOS (*m*/*z*) = 498.90. These peak areas were applied to the appropriate calibration curve to identify the concentration of the analyte in ng/mL, which were then corrected by the dilution factors applied.

### 2.8. Gene Expression Analysis

EpiDermFT tissues were washed with 200 μL of pre-warmed DPBS immediately prior to nucleic acid extraction. EpiDermFT tissues were disrupted and homogenized in 700 μL of QIAzol using a steel bead and TissueLyser II. Homogenates were centrifuged at 15,000× *g* for 10 min at 4 °C. Total RNA was isolated from lysates using the miRNAeasy kit (Qiagen, Germantown, MD, USA) per manufacturer instructions, with a final elution volume of 30 μL per sample. RNA purity and yield were determined on a NanoDrop Spectrophotometer (Thermo Scientific, Waltham, MA, USA). Reverse transcription was performed using the High-Capacity cDNA Reverse Transcription Kit (Applied Biosystems, Foster City, CA, USA) according to the manufacturer’s instructions. TaqMan Fast Universal PCR Master Mix (Applied Biosystems), cDNA, and gene-specific primers (TaqMan Gene Expression Assays) were combined, and real-time quantitative PCR was performed per manufacturer’s instructions. Gene expression was analyzed on a QuantStudio3 (Applied Biosystems) system using cycling conditions recommended by the manufacturer. Relative fold gene expression changes, 2^−ΔΔCt^, were determined compared to each vehicle control (acetone, water, DEGME) and normalized for expression of reference gene *GAPDH* (Hs02786624_g1). Genes evaluated include filaggrin (*Flg*) (Hs00856927_g1), *Flg2* (Hs00418578_m1), loricrin (*Lor*) (Hs01894962_s1), occuludin (*Ocln*) (Hs00170162_m1), *Tslp* (Hs00263639_m1), *S100a8* (Hs00374264_g1), *Il1β* (Hs01555410_m1), *Pparα* (Hs00947536_m1), *Pparγ* (Hs01115513_m1), and *Tnf-α* (Hs00174128_m1).

### 2.9. Cytokine Release

The culture medium was collected from the basal chamber following incubation and frozen at −80 °C. The medium was evaluated with a custom human premixed multi-analyte kit (LXSAHM; R&D Systems, Minneapolis, MN, USA) and analyzed on a MagPix (Luminex, Austin, TX, USA) following the manufacturer’s protocols. Analytes measured included: epidermal growth factor (EGF), S100A8, tumor necrosis factor (TNF)-α, vascular endothelial growth factor (VEGF), transforming growth factor (TGF)-α, and thymic stromal lymphopoietin (TSLP).

### 2.10. Histology

EpiDermFT tissues were washed with 200 μL of pre-warmed DPBS immediately prior to collection. Formalin-fixed paraffin-embedded tissues were sectioned (5 μm) and mounted on slides and stained with hematoxylin and eosin following standard procedures (1 slide/tissue). Slides were brightfield imaged on an Olympus BX63 microscope (Olympus Scientific, Tokyo, Japan). Epidermal thickness was measured from three random views/slide and three measurements/view were taken and averaged.

### 2.11. TEWL Measurements

Transepidermal water loss (TEWL) was determined following 24-h exposure using a VapoMeter (Delfin Technologies, Kuopio, Finland) and an in vitro adapter on EpiDermFT tissues while in the adapter, per the manufacturer’s instructions.

### 2.12. Statistical Analysis

The experimental design consisted of three independent biological replicates; each performed on a separate six-well plate with one tissue per treatment condition per plate. The three replicates were averaged for each treatment combination, yielding *n* = 3 for all statistical analyses, except where a replicate was lost, in which case *n* = 2 is indicated in the corresponding figure legend or table footnote. The use of three independent plate-blocked biological replicates is consistent with established practice for reconstructed human epidermis permeability and barrier studies [39,48]. The EpiDermFT model is a standardized, commercially produced reconstructed human epidermis manufactured under defined quality-control specifications from a consistent source, which minimizes inter-tissue variability. One-way analysis of variance (ANOVA) was conducted for all experiments containing 3 or more groups of the same exposure type. If the ANOVA showed significance at *p* ≤ 0.05, a Dunnett’s multiple comparison test (if comparing to control alone) or Tukey’s multiple comparison test (when comparing all groups) was conducted. Dunnett’s and Tukey’s post-tests control the family-wise error rate within each analysis of variance, so comparisons among concentrations for a given endpoint are corrected for multiple testing. Unpaired t tests were conducted for 2 group comparisons. Normality was assessed by examining the residuals from the ANOVA models using the Shapiro–Wilk test, with normal QQ plots of the residuals inspected for each analysis. Residuals were consistent with a normal distribution (*p* > 0.05). We note that with three replicates per group, formal normality testing has limited power to detect departures from normality. Statistical analysis was conducted using GraphPad Prism (v. 9.3). Results represent the mean ± SD of 3 tissues per group. Statistical significance is designated by * *p* ≤ 0.05, ** *p* ≤ 0.01, and *** *p* ≤ 0.001.

## 3. Results

### 3.1. Vehicle Selection Alters PFAS Permeability Through Reconstructed Human Tissue After 24-h Exposure

**Figure 1:**
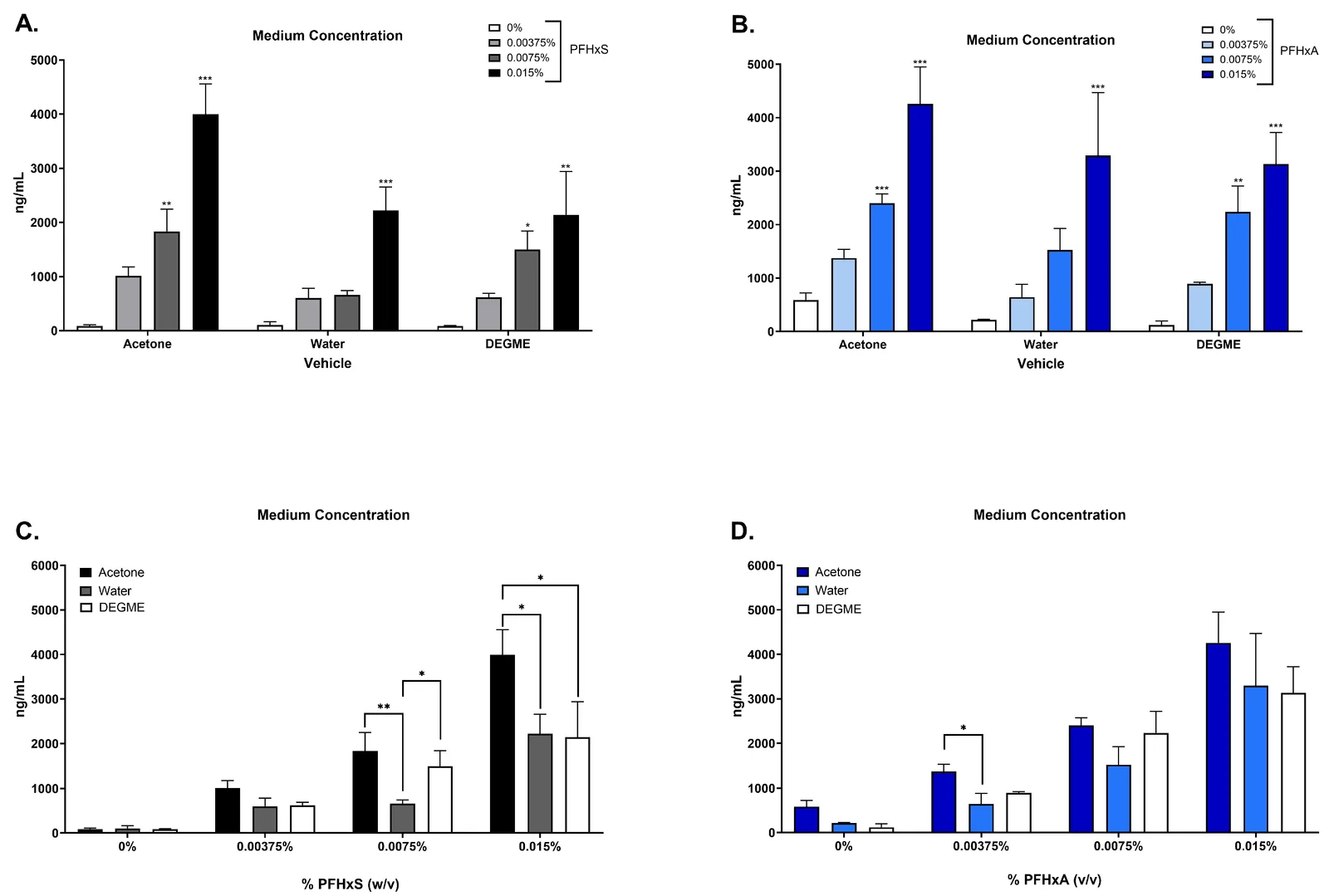
Per- and polyfluoroalkyl substance (PFAS) concentration in the medium after a single 24-h exposure. Concentration of PFAS in the medium after 24-h exposure (0–0.015%) to perfluorohexane sulfonic acid (PFHxS) (**A**,**C**) or perfluorohexanoic acid (PFHxA) (**B**,**D**) dissolved in acetone, water, or diethylene glycol monobutyl ether (DEGME) (**A**–**D**). Bars represent mean [standard deviation ± (SD)] of 3 samples/group (PFHxS 0.00375% acetone and water, PFHxA 0.00375% DEGME *n* = 2). Statistical significance was determined by one-way ANOVA followed by Dunnett’s post-test, relative to 0% control, (**A**,**B**) or Tukey’s post-test (**C**,**D**) as indicated by * *p* < 0.05, ** *p* < 0.01, and *** *p* < 0.001.

For these initial studies, we investigated PFHxS and PFHxA to compare the C6 sulfonic and carboxylic acid compounds. Results from these studies are graphed to compare medium PFAS concentrations in relation to exposure concentrations (Figure 1A,B) and grouped to compare the effect of the vehicle on absorption (Figure 1C,D). Both PFHxS (Figure 1A) and PFHxA (Figure 1B) induced a dose response, with all three vehicles (acetone, water, DEGME) showing a significant increase in PFAS concentration in the medium with an increase in PFAS exposure, indicating that both PFHxS and PFHxA are absorbed through the skin when dissolved in all three vehicles. Both PFHxS and PFHxA concentrations in the medium significantly increased when dissolved in acetone (0.0075% and 0.015%), water (0.015%), and DEGME (0.0075% and 0.015%). When comparing the effect of the vehicle on absorption (Figure 1C,D), the vehicle altered PFAS penetration at 0.0075% and 0.015% PFHxS (Figure 1C) and 0.00375% PFHxA (Figure 1D). When 0.0075% PFHxS was dissolved in water, the PFHxS medium concentration was significantly lower compared to the acetone-vehicle group. With 0.015% PFHxS exposure, both water and DEGME exhibited less penetration (a 44% and 46% decrease, respectively) when compared to acetone (Figure 1C). Vehicle-only significantly affected medium PFHxS concentration in the 0.00375% exposure group, with 53% less PFHxA in the water vehicle group compared to the acetone vehicle group (Figure 1D). A single exposure to either PFHxS or PFHxA for 24 h did not significantly increase LDH levels, suggesting that no cytotoxicity occurred at the concentrations used (Supplemental Figure S1A,B).

**Figure 2:**
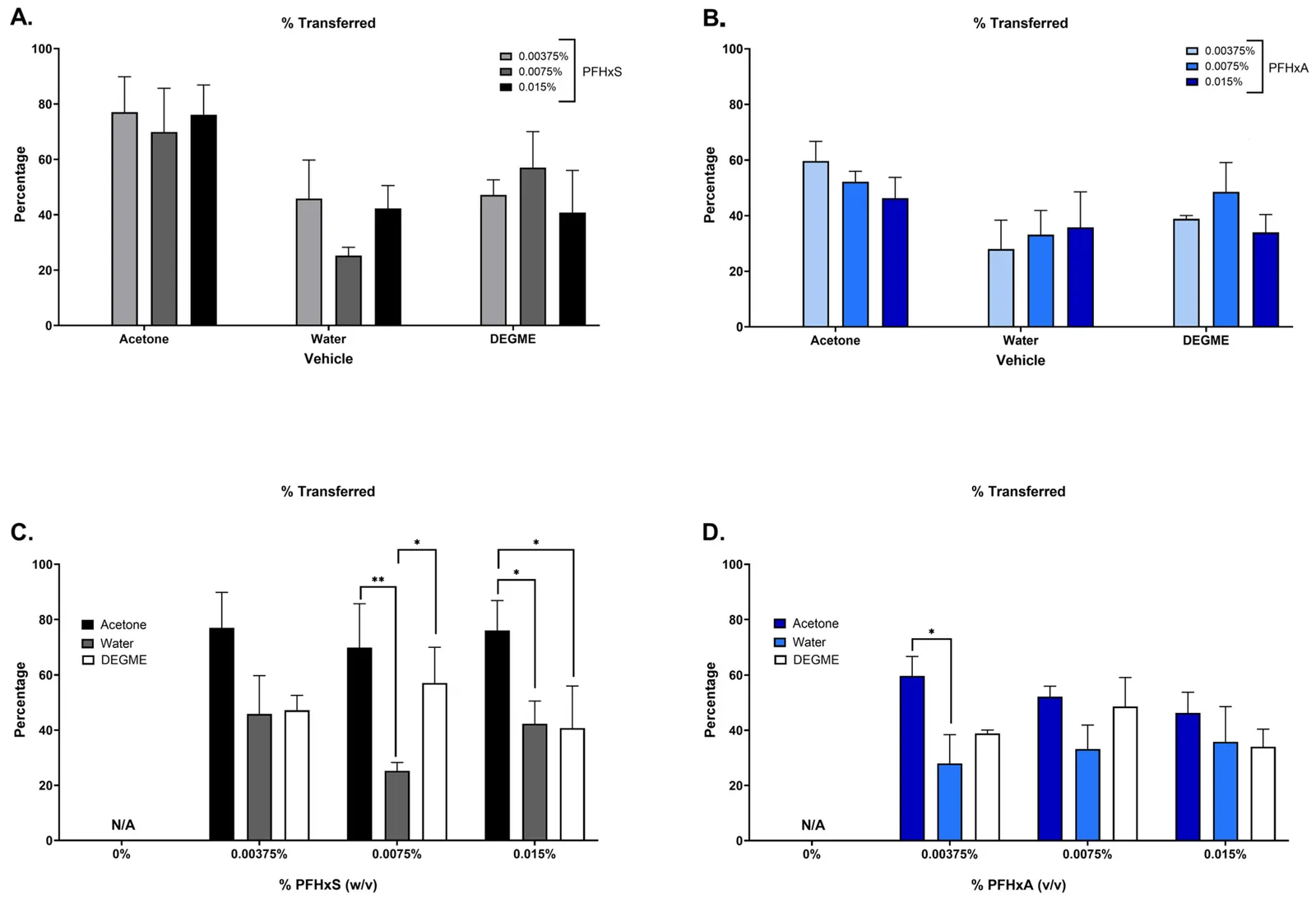
Percentage of PFAS transferred from skin tissue to the medium after a single 24-h exposure. Percentage of PFAS in the medium after 24-h exposure (0–0.015%) to PFHxS (**A**,**C**) or PFHxA (**B**,**D**) dissolved in acetone, water, or DEGME (**A**–**D**). Bars represent mean (± SD) of 3 samples/group (PFHxS 0.00375% acetone and water, PFHxA 0.00375% DEGME *n* = 2), with statistical significance determined by one-way ANOVA followed by Tukey’s post-test as indicated by * *p* < 0.05 and ** *p* < 0.01.

Percent transferred (dose applied to top of tissue compared to recovery concentration in the media) calculations revealed no significant differences due to the dose applied, suggesting that concentration does not influence permeability through the skin with either PFHxS (Figure 2A) or PFHxA (Figure 2B). PFHxS at 0.015% dissolved in acetone showed a 76% transfer from the dose applied to the concentration detected in the medium (Figure 2C), and 0.015% PFHxA showed a 46.3% transfer in acetone (Figure 2D). When dissolved in water, 0.015% PFAS showed a 42.3% (PFHxS) and 35.8% (PFHxA) transfer, and in DEGME a 40.8% (PFHxS) and 34% (PFHxA) transfer (Figure 2D). The percentage transferred of PFHxA was significantly lower than that of PFHxS only, with 0.015% PFHxA in acetone. No significant difference was found between PFHxS and PFHxA at any other concentration with any other vehicle (Supplement Figure S2). Additionally, the PFHxS percent transferred was significantly altered depending on the vehicle (Figure 2C). When compared to acetone, the percent transferred for 0.0075% PFHxS in water was 64% less. While there was no difference between PFHxS in acetone versus DEGME, PFHxS in water was also significantly decreased compared to DEGME (Figure 2C). In water, the 0.015% PFHxS percent transferred decreased by 44% and in DEGME by 46% compared to PFHxS in acetone (Figure 2C). The PFHxA percentage transferred was not altered by vehicle at the middle or highest concentrations of 0.0075% and 0.015%, but PFHxA in water did decrease compared to acetone at 0.00375% by 53% (Figure 2D).

### 3.2. Vehicle Selection Alters PFAS Permeability Through Reconstructed Human Tissue After 4-h Exposure

**Figure 3:**
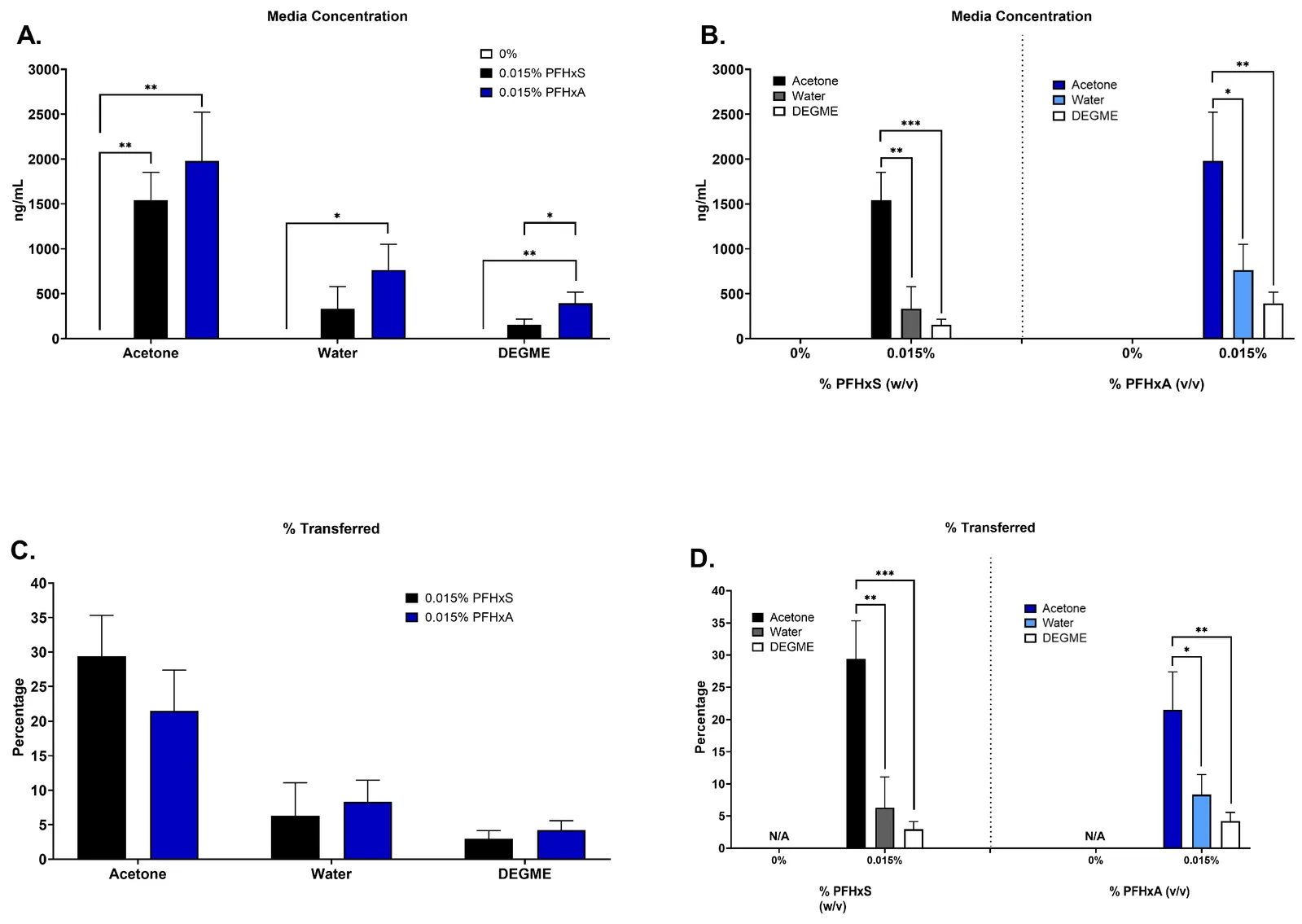
PFAS detection in the medium after a single 4-h exposure. Concentration (**A**,**B**) or percent transferred (**C**,**D**) of PFAS in the medium after 4-h exposure to PFHxS or PFHxA (0.015%) dissolved in acetone, water, or DEGME. Bars represent mean (± SD) of 3 samples/group. Statistical significance was determined by unpaired *t*-test relative to control (**A**), PFHxS vs PFHxA (**C**), or one-way ANOVA followed by Tukey’s post-test (**B**,**D**), as indicated by * *p* < 0.05, ** *p* < 0.01, and *** *p* < 0.001.

An additional study was performed with 4 h of PFAS exposure to investigate the effects of PFAS vehicle following a shorter exposure time. These studies were conducted with only the high concentration (0.015%) of each compound dissolved in all three vehicles. Both PFHxS and PFHxA were absorbed after 4 h of exposure with significant increases in PFAS concentration in the medium when dissolved in acetone and DEGME. With water as the vehicle, only PFHxA was significantly increased (Figure 3A). The ability of both PFHxS and PFHxA to be absorbed through the skin was significantly altered based on vehicle after 4 h of exposure (Figure 3B). PFHxS concentration significantly decreased in the medium after 0.015% exposure when dissolved in water (78% decrease) and DEGME (90% decrease) compared to acetone (Figure 3B). With 0.015% PFHxA, there was also a decrease in medium concentration when dissolved in water (61%) and DEGME (80%) compared to acetone (Figure 3B).

Similar to the 24-h exposure, following 4 h of exposure, the percent transferred calculation revealed no significant difference between PFHxS and PFHxA with any vehicle at 0.015% (Figure 3C). Both PFHxS and PFHxA in water and DEGME had significantly lower values in the medium compared to when dissolved in acetone (Figure 3D). PFHxS dissolved in acetone showed a 29% transfer from the dose applied to the tissue compared to the concentration detected in the medium, and PFHxA showed a 21.5% transfer in acetone. PFAS dissolved in water showed a 6.3% (PFHxS) and 8.3% (PFHxA) transfer and in DEGME it showed a 3% (PFHxS) and 4.2% (PFHxA) transfer (Figure 3D). Collectively, these findings suggest that PFAS can penetrate the skin as soon as 4 h after exposure.

### 3.3. Tissue Retained PFAS Following a 24-h Exposure

**Figure 4:**
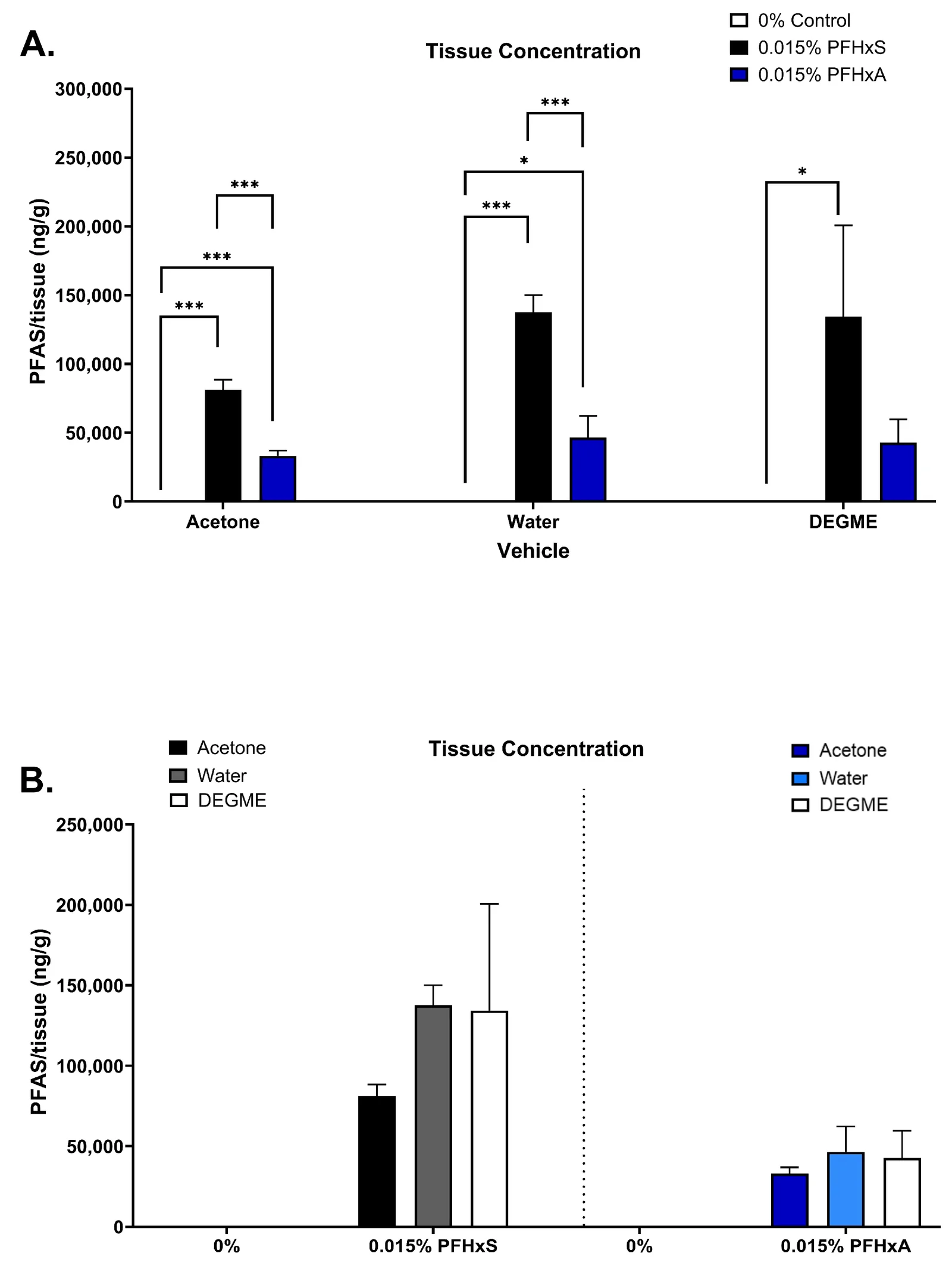
PFAS detection in tissue after a single 24-h exposure. Concentration of PFAS in EpiDermFT tissue after 24-h exposure to PFHxS or PFHxA (0.015%) dissolved in acetone. Bars represent mean (± SD) of 3 samples/group. Statistical significance was determined by unpaired *t*-test relative to control and PFHxS vs. PFHxA (**A**), or one-way ANOVA followed by Tukey’s post-test (**B**) as indicated by * *p* < 0.05 and *** *p* < 0.001. Dotted line separtes PFHxS from PFHxA data.

Both PFHxS and PFHxA induced a significant increase in concentration in the tissue following PFAS exposure. Both PFHxS (in acetone, water, DEGME) and PFHxA (in acetone, water) penetrated the tissue, suggesting that although both compounds are detected in the medium, some of the dose is retained within the tissue. A significantly higher concentration of PFHxS was detected in the tissue, compared to PFHxA, when dissolved in both acetone and water (Figure 4A). When dissolved in DEGME, only PFHxS showed a significant increase in the tissue concentration (compared to control), and there was no significant difference between PFHxS and PFHxA. Also, there was no significant difference in the tissue concentration when comparing the different vehicles with PFHxS or PFHxA exposure (Figure 4B).

### 3.4. Dermal Exposure to PFAS Altered Protein and Gene Expression

Cytokines and growth factors known to be produced by keratinocytes were evaluated at the protein level in the medium following 24-h PFAS exposure. Few changes were observed with PFHxS exposure, having only a decrease in TNF-α at 0.0075% in water and a decrease in TGF-α at 0.0075 and 0.015% in water (Table 1). In contrast, PFHxA exposure produced multiple changes in cytokine production. Two cytokines were consistently altered between all three vehicles: S100a8 and VEGF. S100a8 was significantly increased with PFHxA in acetone at all three concentrations, in water S100a8 was significantly increased at 0.00375 and 0.0075%, and in DEGME at 0.015% (Table 2). VEGF was significantly increased after PFHxA exposure in acetone (0.0075%), water (0.00375, 0.0075, 0.015%), and DEGME (0.0075, 0.015%). Additionally, the significant increases in VEGF with PFHxA were dose-responsive (significant linear trend test) in all three vehicles and in S100a8 in acetone and DEGME. PFHxA also induced significant changes in TNF-α and TGF-α at 0.00375% in acetone and TSLP in water. There were no changes in cytokine levels with PFHxS or PFHxA after 4 h of exposure (Supplemental Figure S3). TNF-α, TSLP, and TGF-α were all near or below the limit of detection after 4-h exposure.

A single dermal exposure to PFHxS for 24 h showed no changes in gene expression (*S100a8*, *Il-1β*, *Tslp*, *Pparα*, *Pparγ*, *Tnf-α*) in any of the three vehicles tested (Table 3). However, one gene was consistently altered with PFHxA in all three vehicles: *Pparγ*. *Pparγ* gene expression decreased with PFHxA dissolved in acetone (0.0075, 0.015%), in water (0.00375, 0.0075, 0.015%), and in DEGME (0.00375, 0.0075, 0.015%) (Table 4). *S100a8* gene expression was increased with PFHxA in water (0.00375, 0.0075%) and in DEGME (0.00375, 0.0075, 0.015%). The significant increases in *Pparγ* with PFHxA were dose-responsive (significant linear trend test) in all three vehicles and in *S100a8* in water and DEGME. After 4 h of exposure, 0.015% PFHxS increased *S100a8* (acetone), Pparα (acetone), and *Pparγ* (acetone) (Supplemental Figure S4). There were no changes in gene expression with PFHxA after 4-h exposure.

### 3.5. Exposure to PFAS Altered Skin Barrier Gene Expression

**Figure 5:**
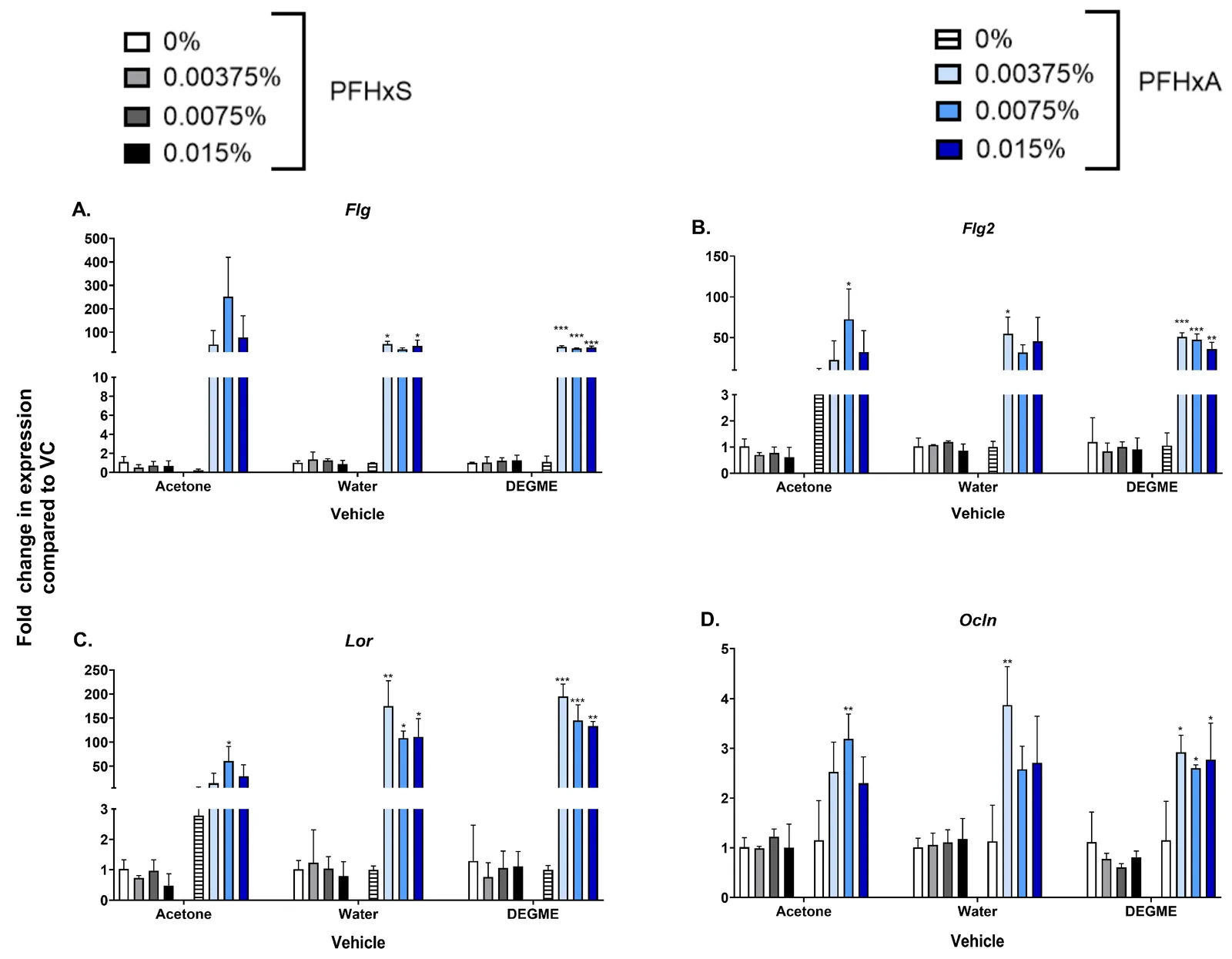
Gene expression following a single 24-h PFAS exposure to EpiDermFT. Fold-change in gene expression compared to vehicle control of (**A**) filaggrin (*Flg*), (**B**) filaggrin2 (*Flg2*), (**C**) loricirin (*Lor*), and (**D**) occludin (*Ocln*) following a single 24-h exposure with PFHxS or PFHxA (0 to 0.015%) (**A**–**D**). Bars represent mean (± SD) of 3 samples/group (PFHxS 0.00375% acetone and water, PFHxA 0.00375% DEGME n = 2). Statistical significance was determined by one-way ANOVA followed by Dunnett’s post-test, relative to 0% control, as indicated by * *p* < 0.05, ** *p* < 0.01, and *** *p* < 0.001.

Dermal toxicity was further evaluated with investigations of skin barrier gene expression in the EpiDermFT tissue. Exposure to PFHxA for 24 h significantly increased expression of *Flg* (Figure 5A), *Flg2* (Figure 5B), *Lor* (Figure 5C), and *Ocln* (Figure 5D) when dissolved in all three vehicles. PFHxA increased *Flg* gene expression by 48-, 42-, and 34-fold, in acetone, water, and DEGME, respectively. *Flg2* gene expression was significantly increased with acetone (0.0075%), water (0.00375%), and DEGME (0.00375, 0.0075, 0.015%). PFHxA in water and DEGME had the largest effect on *Lor* with 0.015% increasing *Lor* gene expression by 29-, 111-, and 133-fold, in acetone, water, and DEGME, respectively. *Ocln* gene expression had the lowest increase of the skin barrier genes but still showed significant increases with acetone (0.0075%), water (0.00375%), and DEGME (0.00375, 0.0075, 0.015%). No effects on skin barrier gene expression were observed with PFHxS in any of the three vehicles (Figure 5). After 4 h of exposure, very slight (under 2-fold) increases were seen with 0.015% PFHxS (in water) on *Flg*, *Flg2*, and *Lor* gene expression (Supplemental Figure S5). *Ocln* was the only gene altered after 4 h of PFHxA exposure when dissolved in DEGME (Supplemental Figure S5).

**Table 1:** Cytokines and growth factors (pg/mL) released following 24 h of PFHxS exposure on EpiDermFT.

	PFHxS (*w*/*v*)											
	Acetone				Water				DEGME			
Cytokine	0%	0.00375%	0.0075%	0.015%	0%	0.00375%	0.0075%	0.015%	0%	0.00375%	0.0075%	0.015%
TNF-α	25.36 ± 0.41	24.24 ± 0.12	25.98 ± 2.03	25.52 ± 1.23	20.09 ± 0.41	18.89 ± 0.58	18.24 ± 0.31 *	19.55 ± 0.39	11.95 ± 1.14	13.78 ± 3.60	11.82 ± 1.98	10.45 ± 0.36
S100a8	478.00 ± 4.00	468.15 ± 29.49	470.36 ± 35.48	446.98 ± 10.02	374.91 ± 17.23	374.93 ± 13.68	358.20 ± 2.01	354.37 ± 14.97	229.19 ± 21.97	356.28 ± 18.21	236.82 ± 14.46	252.90 ± 8.91
VEGF	2575.52 ± 61.13	2756.99 ± 39.66	2773.50 ± 514.12	2353.11 ± 106.81	1937.01 ± 28.81	1841.93 ± 121.54	1685.00 ± 42.55	1653.06 ± 119.96	1749.92 ± 86.58	2145.22 ± 229.83	1881.13 ± 202.68	1918.10 ± 101.60
TSLP	8.56 ± 1.18	7.08 ± 0.03	6.57 ± 0.39	7.96 ± 0.67	5.90 ± 0.31	5.08 ± 1.62	5.02 ± 0.65	4.64 ± 0.35	3.05 ± 0.10	6.08 ± 1.68	3.94 ± 0.62	4.83 ± 1.33
TGF-α	12.68 ± 0.50	13.16 ± 0.72	13.87 ± 0.58	13.39 ± 1.59	11.56 ± 0.49	10.52 ± 0.00	9.80 ± 0.14 **	9.39 ± 0.16 **	8.10 ± 0.00	8.41 ± 2.30	6.94 ± 0.17	7.00 ± 0.44
EGF	141.09 ± 3.27	152.75 ± 12.66	153.51 ± 2.69	144.10 ± 7.62	132.83 ± 4.40	130.40 ± 8.29	125.03 ± 4.24	119.25 ± 4.02	83.62 ± 19.04	72.76 ± 7.57	68.02 ± 2.75	68.52 ± 5.14

Values are expressed as the means (± SE) for each group, *n* = 3 tissues/group (PFHxS 0.00375% acetone and water *n* = 2). Tumor necrosis factor alpha (TNF-α), vascular endothelial growth factor (VEGF), thymic stromal lymphopoietin (TSLP), transforming growth factor alpha (TGF-α), epidermal growth factor (EGF). * *p* < 0.05 and ** *p* < 0.01. Bold denotes significant difference from corresponding 0% control.

**Table 2:** Cytokines and growth factors (pg/mL) released following 24 h of PFHxA exposure on EpiDermFT.

	PFHxA (*v*/*v*)											
	Acetone				Water				DEGME			
Cytokine	0%	0.00375%	0.0075%	0.015%	0%	0.00375%	0.0075%	0.015%	0%	0.00375%	0.0075%	0.015%
TNF-α	10.06 ± 0.47	13.51 ± 0.77 **	11.04 ± 0.17	11.81 ± 0.28	8.06 ± 0.84	11.94 ± 0.23	12.08 ± 1.38	10.45 ± 1.30	3.28 ± 0.64	5.32 ± 1.04	6.26 ± 1.19	7.37 ± 1.71
S100a8	182.60 ± 7.39	256.28 ± 13.29 **	267.28 ± 1.69 **	241.05 ± 15.67 *	160.54 ± 11.88	235.12 ± 14.44 *	251.20 ± 19.90 **	218.19 ± 11.10	116.83 ± 1.87	139.01 ± 15.46	138.10 ± 2.78	149.62 ± 5.53 *
VEGF	711.11 ± 250.78	1295.28 ± 188.17	2132.89 ± 91.69 **	1509.96 ± 309.45	651.89 ± 147.62	1421.08 ± 207.78 *	1534.60 ± 160.61 *	1433.59 ± 216.37 *	2465.33 ± 847.63	4684.85 ± 364.66	4769.93 ± 188.97 *	4976.03 ± 144.87 *
TSLP	4.06 ± 0.21	7.82 ± 1.35	6.05 ± 1.04	6.11 ± 0.93	2.49 ± 0.17	14.52 ± 1.45 **	6.64 ± 1.41	7.35 ± 2.55	1.28 ± 0.19	7.98 ± 4.27	4.76 ± 1.39	7.96 ± 1.64
TGF-α	6.67 ± 0.29	8.75 ± 0.76 *	7.44 ± 0.38	7.22 ± 0.22	5.45 ± 0.66	7.22 ± 0.54	7.43 ± 0.76	7.22 ± 0.79	2.89 ± 0.00	2.89 ± 0.71	2.66 ± 0.29	7.43 ± 3.67
EGF	41.60 ± 8.37	58.97 ± 5.51	62.30 ± 3.51	56.91 ± 2.34	63.48 ± 4.79	59.87 ± 2.99	64.77 ± 3.95	56.27 ± 3.73	74.85 ± 2.84	70.57 ± 1.50	71.00 ± 1.03	69.30 ± 3.77

Values are expressed as the means (± SE) for each group, *n* = 3 tissues/group (PFHxA 0.00375% DEGME *n* = 2). * *p* < 0.05 and ** *p* < 0.01. Bold denotes significant difference from corresponding 0% control.

**Table 3:** Gene expression (fold change in expression) following 24 h of PFHxS exposure on EpiDermFT.

	PFHxS (*w*/*v*)											
	Acetone				Water				DEGME			
Gene	0%	0.00375%	0.0075%	0.015%	0%	0.00375%	0.0075%	0.015%	0%	0.00375%	0.0075%	0.015%
*S100a8*	1.01 ± 0.11	1.17 ± 0.31	0.76 ± 0.10	0.62 ± 0.15	1.06 ± 0.27	0.84 ± 0.02	0.86 ± 0.13	1.26 ± 0.19	1.01 ± 0.12	1.28 ± 0.30	1.17 ± 0.19	1.30 ± 0.15
*Il-1β*	1.23 ± 0.45	1.11 ± 0.63	0.88 ± 0.20	0.90 ± 0.34	1.13 ± 0.36	2.68 ± 2.40	1.00 ± 0.12	1.96 ± 0.44	1.28 ± 0.62	1.35 ± 0.87	0.78 ± 0.35	0.99 ± 0.23
*Tslp*	1.06 ± 0.25	0.99 ± 0.36	0.97 ± 0.12	0.89 ± 0.07	1.04 ± 0.21	1.21 ± 0.61	0.82 ± 0.06	1.24 ± 0.13	1.01 ± 0.12	1.07 ± 0.19	1.14 ± 0.05	1.57 ± 0.25
*Pparα*	1.01 ± 0.09	1.07 ± 0.02	1.00 ± 0.06	1.12 ± 0.10	1.03 ± 0.17	1.06 ± 0.18	0.89 ± 0.07	1.30 ± 0.13	1.04 ± 0.20	0.89 ± 0.06	0.94 ± 0.05	1.24 ± 0.25
*Pparγ*	1.02 ± 0.13	1.11 ± 0.08	1.20 ± 0.27	1.38 ± 0.25	1.01 ± 0.07	1.11 ± 0.09	0.93 ± 0.10	1.19 ± 0.09	1.03 ± 0.22	1.04 ± 0.03	1.06 ± 0.11	1.13 ± 0.11
*Tnf-α*	1.00 ± 0.01	1.54 ± 0.53	0.91 ± 0.17	2.76 ± 0.87	1.04 ± 0.21	0.76 ± 0.25	0.83 ± 0.19	1.53 ± 0.28	1.09 ± 0.36	0.78 ± 0.29	0.54 ± 0.04	0.64 ± 0.04

Values are expressed as the means (± SE) for each group, *n* = 3 tissues/group (PFHxS 0.00375% acetone and water *n* = 2). Interleukin-1β (*Il-1β*), peroxisome proliferator-activated receptor alpha (*Pparα*), peroxisome proliferator-activated receptor gamma (*Pparγ*).

**Table 4:** Gene expression (fold change in expression) following 24 h of PFHxA exposure on EpiDermFT.

	PFHxA (*v*/*v*)											
	Acetone				Water				DEGME			
Gene	0%	0.00375%	0.0075%	0.015%	0%	0.00375%	0.0075%	0.015%	0%	0.00375%	0.0075%	0.015%
*S100a8*	1.72 ± 1.40	3.85 ± 2.42	6.06 ± 2.31	3.27 ± 0.86	0.97 ± 0.14	20.48 ± 3.34 *	22.34 ± 6.23 *	13.95 ± 2.29	1.00 ± 0.04	29.52 ± 2.72 ***	28.13 ± 1.62 ***	18.14 ± 1.93 **
*Il-1β*	1.19 ± 0.47	1.48 ± 0.37	0.81 ± 0.20	1.02 ± 0.22	1.18 ± 0.41	11.98 ± 1.75 *	5.28 ± 2.72	6.76 ± 2.86	1.02 ± 0.14	4.60 ±3.85	3.41 ± 1.40	5.97 ± 2.25
*Tslp*	1.07 ± 0.29	1.74 ± 0.30	2.56 ± 1.01	1.43 ± 0.10	1.09 ± 0.33	5.66 ± 0.87 *	4.03 ± 1.16	3.82 ± 0.87	1.17 ± 0.47	3.02 ± 1.13	3.41 ± 0.82	4.02 ± 0.57
*Pparα*	1.09 ± 0.16	1.05 ± 0.05	0.97 ± 0.23	0.75 ± 0.07	1.00 ± 0.02	1.80 ± 0.10 *	1.39 ± 0.25	1.26 ± 0.15	1.00 ± 0.05	1.35 ± 0.24	1.28 ± 0.10	1.27 ± 0.14
*Pparγ*	1.01 ± 0.07	0.91 ± 0.05	0.56 ± 0.13 *	0.56 ± 0.15 *	1.03 ± 0.15	0.63 ± 0.07 *	0.59 ± 0.06 *	0.45 ± 0.07 **	1.01 ± 0.12	0.57 ± 0.06 **	0.67 ± 0.08 *	0.49 ± 0.01 **
*Tnf-α*	1.02 ± 0.13	2.61 ± 0.60	1.25 ± 0.32	1.72 ± 0.62	1.00 ± 0.04	18.15 ± 2.01 *	14.92 ± 3.25 *	13.18 ± 4.00	1.30 ± 0.83	2.47 ± 0.45	4.07 ± 1.49	3.25 ± 0.33

Values are expressed as the means (± SE) for each group, *n* = 3 tissues/group (PFHxA 0.00375% DEGME *n* = 2). * *p* < 0.05, ** *p* < 0.01, and *** *p* < 0.001. Bold denotes significant difference from corresponding 0% control.

### 3.6. Exposure to PFAS Disrupts Skin Barrier Integrity

**Figure 6:**
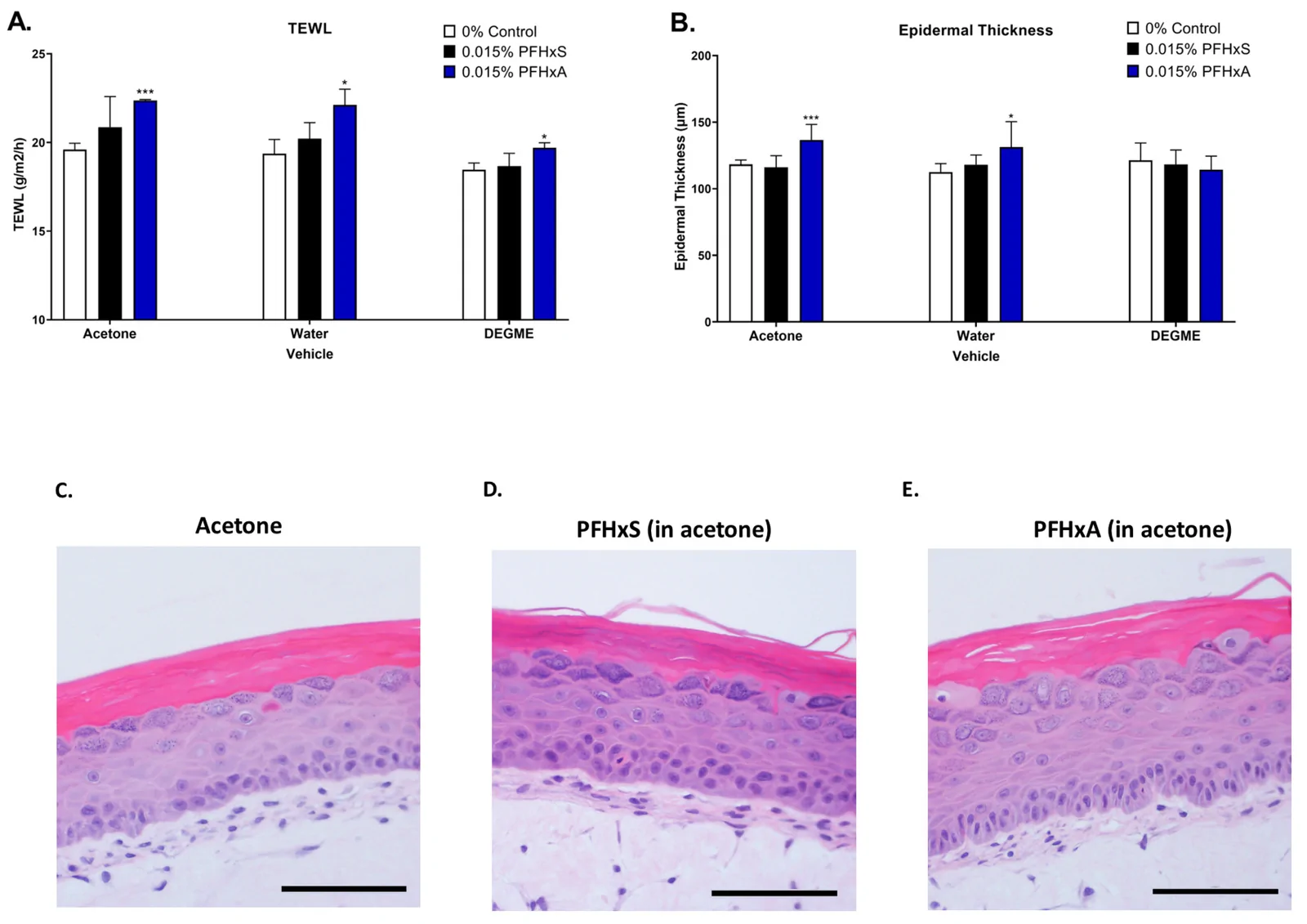
PFAS exposure alters epidermis on EpiDermFT tissues. Transepidermal water loss (TEWL) of EpiDerm full thickness tissues (**A**) and total epidermal thickness (µm) (**B**) after 24-h of 0.015% PFHxS or PFHxA exposure. Bars represent mean (± SD) of 3 samples/group. Statistical significance was determined by unpaired *t*-test, relative to control and vehicle-dependent effects were evaluated by two-way ANOVA (treatment × vehicle) followed by Tukey’s multiple comparisons test, as indicated by * *p* < 0.05 and *** *p* < 0.001. Representative hematoxylin and eosin images of EpiDermFT tissues following 24-h of exposure to acetone vehicle (**C**), 0.015% PFHxS in acetone (**D**), or 0.015% PFHxA in acetone (**E**). Scale bar = 100 µm.

TEWL measures skin barrier integrity [49] and has been successfully used in HRE models [48]. TEWL significantly increased with 0.015% PFHxA exposure dissolved in all three vehicles after a single 24-h exposure, compared to control (Figure 6A). No increase in TEWL after 0.015% PFHxS exposure dissolved in any of the three vehicles was observed. Transepidermal water loss is reported as absolute values (g/m^2^/h; Figure 6A). Vehicle-control tissues exhibited a baseline TEWL of 19.6 ± 0.4 g/m^2^/h (acetone), 19.4 ± 0.8 g/m^2^/h (water), and 18.5 ± 0.4 g/m^2^/h (DEGME), which serves as the in-model reference values. Additionally, total epidermal thickness also increased with 0.015% PFHxA exposure when dissolved in acetone and water (not DEGME), and PFHxS exposure showed no changes in total epidermal thickness with any of three vehicles (Figure 6B–E). To test whether the barrier effects of each PFAS depended on the vehicle, we conducted two-way ANOVAs (treatment × vehicle) with Tukey’s multiple comparisons test for epidermal thickness and TEWL, examining the effect of vehicle within each PFAS treatment. For epidermal thickness, the vehicle-only controls and PFHxS showed no significant differences across vehicles, whereas PFHxA-induced thickening was vehicle-dependent, being significantly greater in acetone and water than in DEGME. For TEWL, DEGME likewise produced significantly lower responses than acetone. These between-vehicle comparisons indicate that vehicle modulates the magnitude of the barrier response, with acetone, the highest-permeability vehicle in our study, producing the largest effects.

## 4. Discussion

While ingestion has historically been considered the dominant PFAS exposure pathway, growing evidence highlights the need to better characterize dermal absorption and its contribution to systemic dose [10,11,50,51]. A wide range of chemical structures exist under the definition of PFAS, which provides opportunities to prioritize PFAS individually or by defined categories according to their chemical structure, physical-chemical properties, toxic effects, occurrence, use, or other characteristics. Research is needed to uncover the mechanisms of action and compare dermal absorption of PFAS across varying chain lengths and functional groups to inform risk assessment, exposure mitigation strategies, and toxicological understanding. This study advances this area by evaluating the dermal permeability and biological responses of two structurally distinct C6 PFAS, PFHxA (carboxylate) and PFHxS (sulfonate), using a reconstructed human epidermis model, with particular emphasis on disentangling the roles of vehicle and chemical structure. The findings suggest that while vehicle conditions can modulate dermal uptake, intrinsic chemical properties, including functional group identity, are more important determinants of PFAS ability to alter the skin barrier.

Previous work conducted in our laboratory evaluated the systemic toxicity of PFAS (sulfonic acids and carboxylic acids; C4–C8) in mice following dermal exposure, and identified unique differences, suggesting the potential for different mechanisms of action [37,38,52,53,54]. In this study, both PFHxA and PFHxS were demonstrated to penetrate the skin as early as 4 h after exposure with increasing accumulation in the receptor fluid at 24 h. In general, comparable levels of PFHxA and PFHxS were detected at both the 4- and 24-h time points. However, at 24 h, much higher levels of PFHxS were detected in the tissue compared to PFHxA. This finding is consistent with our previous findings conducted in mice [37,38] and epidemiological studies demonstrating a longer half-life for PFHxS (4.7–35 years) [3] compared to PFHxA (32 days) [23].

The incorporation of PFAS into personal-care products, together with occupational contact, most notably firefighting, where AFFFs and contaminated protective equipment create substantial dermal exposure [19,55], has increased the relevance of the dermal route, often alongside co-solvents that may alter solubility and permeability [56]. These foams contain different vehicles compared to environmental exposure that could alter permeability. In the previously mentioned series of in vivo mouse studies conducted in our laboratory, acetone was used as the vehicle control due to solubility and for historical consistency. However, studies have demonstrated that acetone may influence the ionization state of the legacy PFAS, perfluorooctanoic acid (PFOA), thus potentially enhancing penetration through the skin [9]. Therefore, in addition to permeability, the influence of vehicle selection was included as part of this study. Both PFHxS and PFHxA, prepared in all 3 vehicles, were demonstrated to penetrate the skin. The highest levels of permeability were identified when acetone was used as the vehicle at both timepoints, suggesting that acetone causes more rapid absorption of both PFHxS and PFHxA through the skin compared to both water and DEGME. Because acetone is not a realistic human dermal exposure vehicle, the higher permeability observed with acetone should be regarded as a mechanistic benchmark rather than an exposure estimate.

The concentrations used in these studies were selected for hazard identification and to characterize structure- and vehicle-dependent permeability and barrier responses across a measurable dose response rather than to reproduce any single human-exposure scenario. They exceed the PFAS levels typically reported in consumer cosmetics [57], but fall within the range of individual PFAS concentrations reported in undiluted AFFF concentrates, where individual and total PFAS have been measured from the mg/L to g/L range [58]. These applied concentrations are more representative of concentrated occupational contact than of routine consumer product use. Because the EpiDermFT model measures transport from an applied surface dose rather than steady-state internal burden, the media and tissue concentrations reported here are not directly comparable to human serum concentrations.

A recent study evaluated the skin permeability of PFOA, PFHxA, and perfluorobutanoic acid (PFBA) solubilized in acetone or artificial perspirant using an in vitro flow model utilizing porcine skin [59]. The authors found that all PFAS penetrated the skin, and while the carbon chain length affected permeability (longer carbon chain had less permeability), the dosing vehicle did not. However, there were high levels detected in the skin, similar to what we observed, highlighting concerns related to skin retention. Another study looking at dermal penetration of PFAS observed that, while permeation of long-chain PFAS was reduced by the skin barrier, certain short-chain PFAS, such as FBSA, can penetrate human skin in vitro [60]. While the recently referenced studies support the findings described in this paper, our objective is unique in that it also evaluates permeability with respect to the functional group of PFAS with the same carbon chain length.

While both C6 PFAS penetrated the skin, unique biological findings were discovered. To understand the mechanisms underlying dermal penetration and subsequent systemic toxicity seen in the in vivo studies, skin barrier integrity and cytokines and growth factors related to immune response and skin barrier function were evaluated. While significant systemic (altered spleen, liver, and thymus weights) and immune toxicity (decrease in spleen IgM response to sheep red blood cells) were observed in mice [38], few changes in protein expression were observed following in vitro tissue PFHxS exposure. Despite the high PFHxS levels detected in the tissue, only slight decreases in TNF-α at 0.0075% and TGF-α at 0.0075 and 0.015% cytokine expression in water were observed. No changes were observed in gene expression after 24 h in any vehicle, and only isolated increases in *S100a8*, *Pparα*, and *Pparγ* were detected at 4 h in acetone (Supplemental Figure S4). Together, these scattered, non-dose-responsive changes are most likely lacking biological significance and stand in clear contrast to the consistent, dose-responsive effects of PFHxA. This is in contrast to the changes observed in the 28-day mouse study, which demonstrated numerous changes in gene expression in the exposure-site skin [*Il-6*, *Tslp*, *Cxcl1*, *Serpine1*, *S100a8*, *Pparδ* (increase), and *Pparγ* (decrease)] [38].

For PFHxA, two cytokines were consistently altered between all three vehicles: S100a8 and VEGF. Gene expression of *S100a8* was also increased with water and DEGME, and although elevated, statistical significance was not reached when acetone was used as the vehicle. Similar findings were observed in the 28-day animal study; *S100a8* gene expression increased at the dermal exposure site with a decrease in *Pparγ* [37]. Keratinocytes are a major source of S100 proteins, which are involved in many cellular processes, including inflammation. When overexpressed, they can promote pro-inflammatory mediators, immune cell infiltration, and tissue remodeling. Vascular endothelial growth factors (VEGF) are also secreted by keratinocytes in response to inflammation and for wound repair. These functional roles support the observed changes in cytokines and skin barrier integrity for PFHxA.

The most interesting differences between the two PFAS were identified when the skin barrier was evaluated. PFHxA significantly increased expression of all skin barrier genes evaluated with substantial increases in *Flg* (34–77-fold), *Flg2* (36–72-fold), and *Lor* (29–133-fold), respective to vehicle. No changes in skin barrier gene expression were observed for PFHxS. However, elevations in *Lor* and *Flg* were observed for both PFAS in the 28-day mouse study [38]. Consistent with skin barrier data, PFHxA had significant increases in TEWL (all vehicles) and epidermal thickness (acetone and water). No changes were observed for PFHxS. In our previous studies, PFHxS was the only PFAS investigated (out of 8) in a mouse model that showed no histological changes in the ear at the site of exposure. However, PFHxA exposure resulted in hyperplasia, hyperkeratosis, inflammation, and fibrosis. Large transcriptional fold-changes measured at 24 h need not translate into proportional structural remodeling within the same time frame, and the in vitro gene-expression changes reported here are best viewed as early, upstream indicators of barrier perturbation. Together, these findings suggest that PFHxA disrupts skin barrier homeostasis and induces a pro-inflammatory microenvironment. While these early upstream responses, along with potential microenvironments driving downstream responses, can be identified, specific systemic targets cannot be identified based on the results obtained in these studies.

Taken together, the data support a single, coherent interpretation. Across all three vehicles, the carboxylate PFHxA produced consistent, dose-responsive induction of skin-barrier genes (*Flg*, *Flg2*, *Lor*, *Ocln*), altered cytokine release (notably S100a8 and VEGF), and increased TEWL and epidermal thickening, whereas the more highly retained sulfonate PFHxS did not. Vehicle selection modulated the magnitude of transport (acetone > water ≈ DEGME) but did not alter this biological divergence. The determinant of barrier toxicity in this model is therefore the compound’s functional group rather than the vehicle or the internal tissue concentration.

A key question generated from these studies is why PFHxA is more toxic than PFHxS in the in vitro model, especially given the high amount of skin retention for PFHxS. These differences may be influenced by study limitations (e.g., absence of microbiome, vasculature, and immune cells) and the relatively short exposure duration. In general, information related to these chemical and structural characteristics of PFAS is lacking and often based on computational analyses [61]. PFAS are classified based on functional groups, and both sulfonates and carboxylates are classified as anionic (contains one or more acidic functional groups such as carboxylic acids, sulfonic acids, sulfates, and phosphates) and can release a hydrogen ion, thereby forming an anion. These functional groups govern many fate and transport properties of PFAS, and the physicochemical properties could potentially translate into different mechanisms of toxicity. In general, sulfonic acids are stronger than carboxylic acids due to their structural differences and the electron-withdrawing effects of the sulfonyl group, which is supported by the longer half-life and increased skin retention. It should be noted that we measured total PFAS in the tissue and did not separate the freely available fraction from what was bound to protein or lipid, nor did we directly assess receptor binding or metabolism, so these mechanisms are inferred from the existing literature. Even so, the fact that PFHxS accumulated to a greater degree than PFHxA while producing fewer biological changes indicates that the amount retained in the skin does not predict toxicity. This suggests that toxicological outcomes are not solely driven by internal dose, but by compound-specific biological interactions.

In addition to roles in inflammation and wound repair, keratinocytes are dependent on a specific lipid matrix, which helps to support numerous biological processes. Epidemiological and mechanistic research indicate that PFAS exposure can disrupt lipid metabolism by binding to lipid transport proteins, activation of lipid-sensitive nuclear receptors (peroxisome proliferator-activated receptors (PPARs)—as identified in the studies described in this manuscript), and alterations in fatty acid uptake and synthesis, leading to abnormal lipid accumulation, oxidative stress, and pro-oncogenic signaling. Studies have found that sulfonic acid-containing PFAS are generally more lipophilic than carboxylic acid-containing PFAS of the same carbon chain length [62,63,64]. Longer skin retention times resulting in dysregulated lipid metabolism (lipid homeostasis) or other novel receptor binding could ultimately be responsible for the systemic toxicological effects identified in vivo [65]. While these associations were not directly examined in this study, the findings highlight the need to consider all data when making conclusions about toxicity and regulation.

Several limitations of the reconstructed human epidermis model should be considered when interpreting these findings. Although the EpiDermFT model reproduces the stratified, differentiated architecture and barrier function of human epidermis, it lacks a vascular network, resident and infiltrating immune cells, a microbiome, xenobiotic-metabolizing enzymes present in intact skin, and systemic feedback. The exposure durations examined (4 and 24 h) capture only acute responses and cannot address chronic or repeated exposure. In addition, the use of three replicates of a standardized model favors internal consistency but does not capture population variability. Further confirmation of these findings in excised human skin from multiple donors is needed. Consequently, the endpoints measured here reflect local, upstream keratinocyte-driven responses, and extrapolation to systemic toxicity or long-term outcomes require corroborating in vivo data. Additionally, it is important to define the scope of these findings. As an in vitro RHE study, this study contributes to hazard identification and to understanding structure–activity relationships among PFAS; it does not quantify the fractional contribution of the dermal route to human PFAS exposure or estimate absorbed-dose risk. Translating these observations into exposure or risk assessment would require quantitative human dermal exposure data together with absorption and pharmacokinetic modeling that are beyond the scope of this study and model. While these data establish that both compounds penetrate the skin and characterize relative dermal hazard and structure–activity behavior, the absorbed dose and its systemic risk cannot be estimated from an in vitro model of this kind. The value of this study lies in demonstrating that two C6 PFAS of identical chain lengths but differing functional groups produce markedly different barrier responses, a distinction directly relevant to how PFAS are prioritized and grouped for assessment. Additionally, the use of separate extraction methods for the medium and tissue preclude a closed mass balance, which should be considered when interpreting the absolute recovery values. There is also the potential for PFAS loss to adsorption to plastic, evaporation, or volatility of the vehicle, and the absence of a collected surface wash.

High-quality scientific data to better understand PFAS exposure pathways and toxicological mechanisms are an important area of research to inform federal decisions that reduce risks to human health and the environment [66]. The findings reported in this manuscript demonstrate that PFAS can be absorbed through the skin when applied in different vehicles. Functional group-dependent toxicity, rather than vehicle-driven permeability, appears to be a primary determinant of dermal PFAS effects. The differential data for PFHxA and PFHxS generated in both in vitro and in vivo highlight the critical need to consider all factors when developing regulatory recommendations.

## 5. Conclusions

This study demonstrates that two structurally distinct C6 PFAS, the carboxylate PFHxA and the sulfonate PFHxS, both penetrate a reconstructed human epidermis model as early as 4 h after a single topical exposure, with continued accumulation by 24 h. Vehicle selection modulated the magnitude of transport, with acetone producing higher permeability than water and DEGME vehicles, but it did not alter the pattern of biological response. Despite substantially greater tissue retention, PFHxS produced only scattered, non-dose-responsive changes and no effect on barrier integrity. Meanwhile, PFHxA elicited consistent, dose-responsive induction of skin-barrier genes, altered release of inflammation- and repair-associated mediators, increased TEWL, and epidermal thickening across vehicles. These findings indicate that dermal barrier toxicity in this model is governed by the compound’s functional group rather than the vehicle or the amount retained in the tissue and suggest that internal dose alone does not predict biological effect. The demonstration that two PFAS of similar chain lengths but differing functional groups produce markedly different barrier responses suggests that functional-group-dependent toxicity should be incorporated into how structurally related PFAS are grouped and prioritized for regulatory assessment.

## Data Availability

The original data presented in the study are openly available in NIOSH Data and Statistics Gateway at www.cdc.gov/niosh/data (accessed on 18 June 2026).

## Supplementary Materials

The following supporting information can be downloaded at: https://www.mdpi.com/article/10.3390/toxics14090756/s1, Figure S1: LDH released from EpiDerm tissues; Figure S2: Percentage of PFAS transferred from skin tissue to receptor fluid after a single 24-h exposure; Figure S3: Cytokines and growth factors (pg/mL) released following a single 4-h PFAS exposure on EpiDerm; Figure S4: Gene expression following a single 4-h PFAS exposure to EpiDerm; Figure S5: Gene expression following a single 4-h PFAS exposure to EpiDerm.

## Abbreviations

The following abbreviations are used in this manuscript:

AFFFs	Aqueous film-forming foams
ALI	Air–liquid interface
ANOVA	One-way analysis of variance
DEGME	Diethylene glycol monobutyl ether
DMEM	Dulbecco’s Modified eagle’s medium
DPBS	Dulbecco’s phosphate-buffered saline
PFAS	Per- and polyfluoroalkyl substances
PFHpS	Perfluoroheptanesulfonic acid
PFHxA	Perfluorohexanoic acid
PFHxS	Perfluorohexane sulfonate
PFOS	Perfluorooctanesulfonic acid
PFPeA	Perfluoropentanoic acid
RHE	Reconstructed human epidermis
TEWL	Transepidermal water loss
