# Supplementary material for: Dermal Permeability of Perfluorohexane Sulfonic Acid (PFHxS) and Perfluorohexanoic Acid (PFHxA) in Varying Vehicles and the Effects on Skin Integrity in a Reconstructed Human Epidermis Model
Source: Toxics. 2026 Aug 26;14(9):756. doi: 10.3390/toxics14090756 (PMC13611144; doi:10.3390/toxics14090756)
Supplement: Supplementary file 1 [file toxics-14-00756-s001.zip › toxics-4481091-Supplementary.pdf]

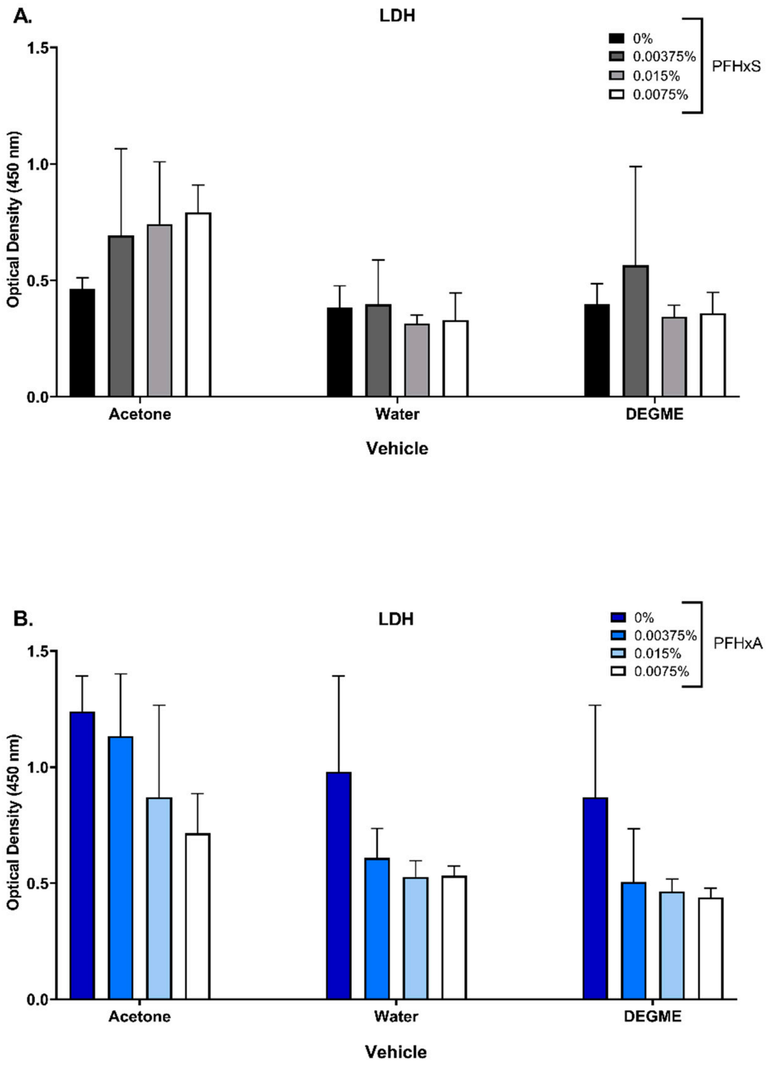

Supplemental Figure S1. LDH released from EpiDerm tissues. Culture media (10 µL) was collected after 24 h of the single exposure and transferred to a clear flat-bottomed 96-well plate. The LDH-Cytotoxicity Colorimetric Assay Kit II (BioVision, Milpitas, CA, USA) was used to perform the lactate dehydrogenase (LDH) release assay per manufacturer instructions. Absorbance (at 450 nm) was measured in duplicate for each sample using a Varioskan Lux (Thermo Scientific, Waltham, MA, USA) microplate spectrophotometer. Values were compared to vehicle control. LDH released into supernatants of EpiDerm tissue after a single 24-hour exposure to PFHxS (A) or PFHxA (B) (0 - 0.015%) dissolved in acetone, water, or DEGME (A, B). Bars represent mean (± SD) of 2-3 samples/group.

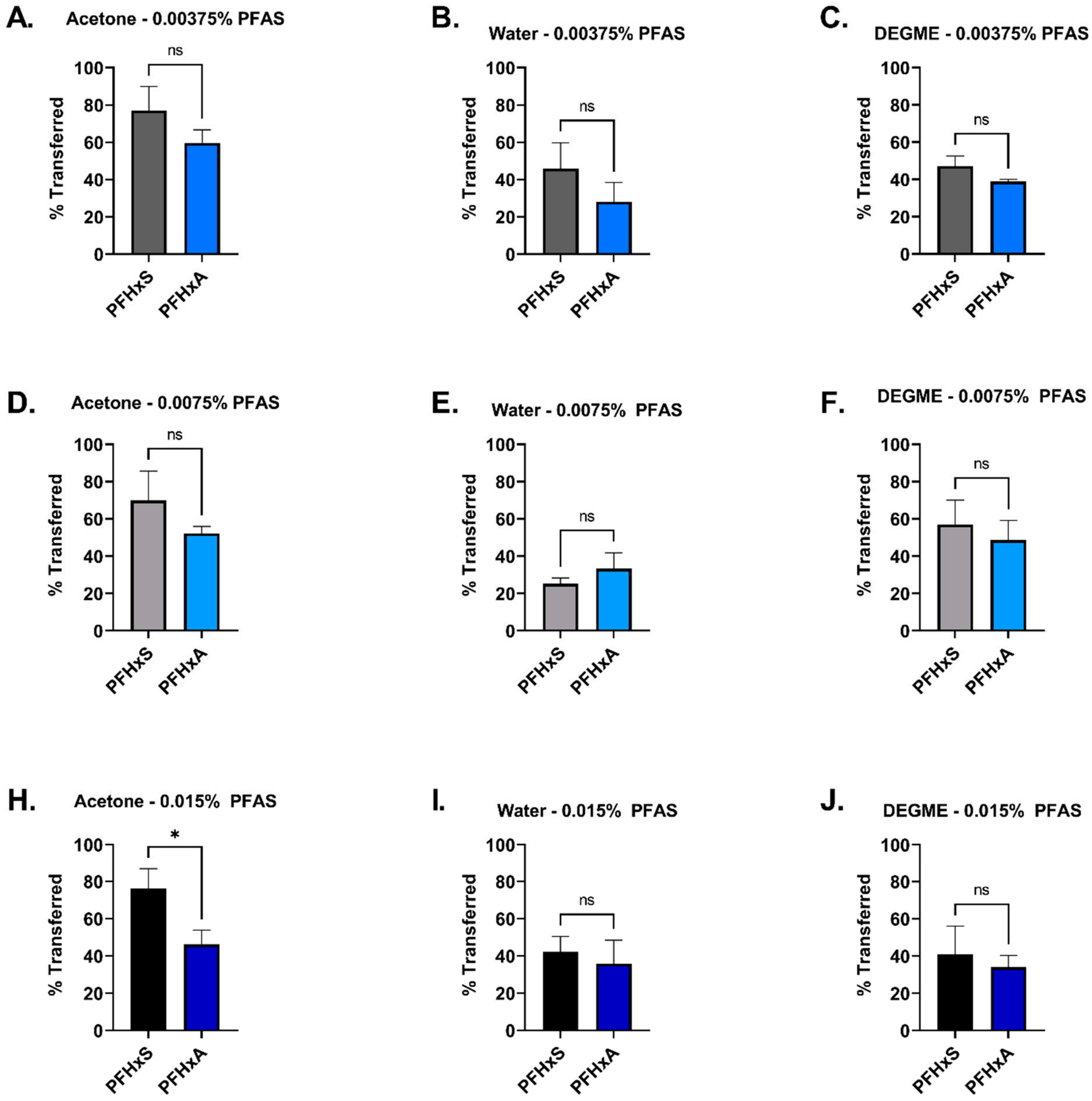

Supplemental Figure S2. Percentage of PFAS transferred from skin tissue to receptor fluid after a single 24-hour exposure. Percentage of PFAS in receptor fluid after 24-hour exposure to PFHxS or PFHxA at 0.00375% (A-C), 0.0075% (D-F) or 0.015% (H-J) dissolved in acetone (A, D, H), water (B, E, I), or DEGME (C, F, J). Bars represent mean (± SD) of 2-3 samples/group, with statistical significance indicated as $*p < 0.05$.

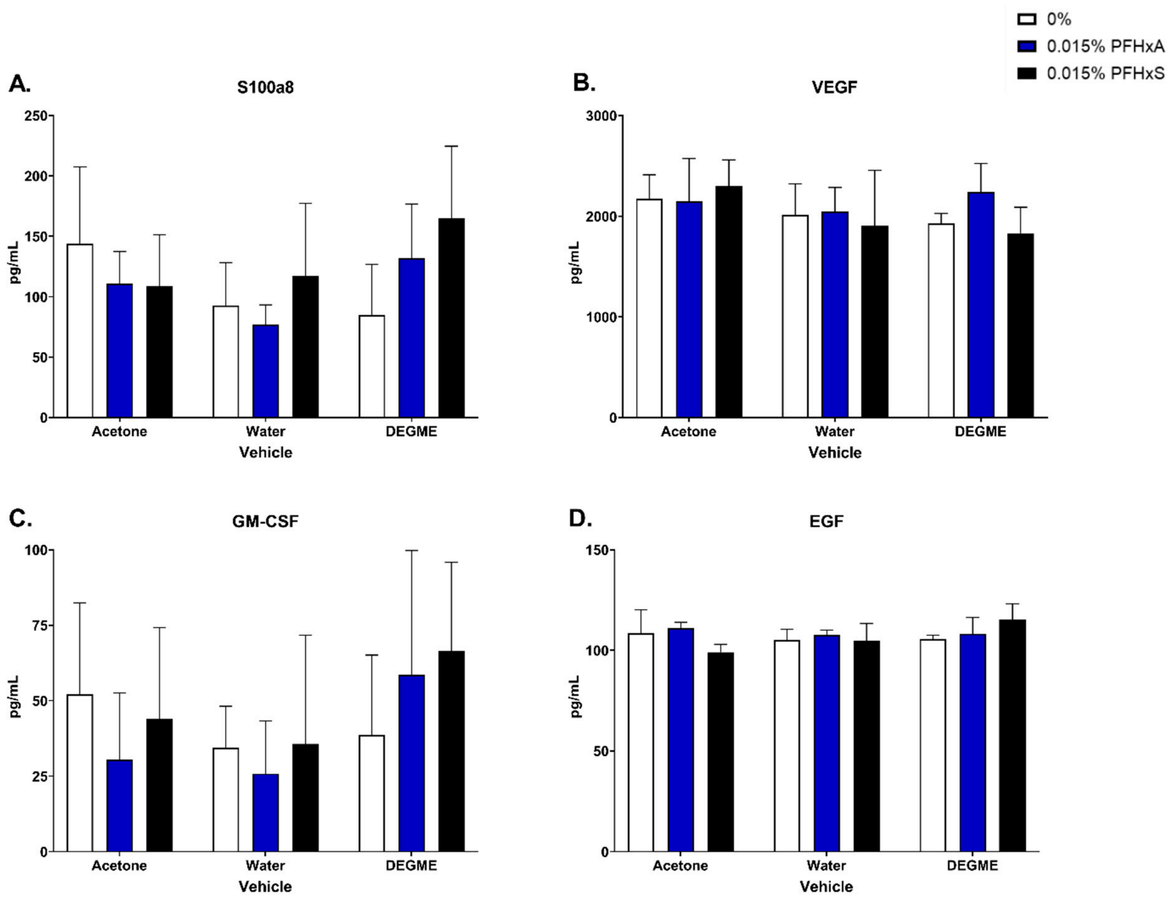

Supplemental Figure S3. Cytokines and growth factors (pg/mL) released following a single 4-hour PFAS exposure on EpiDerm. Concentrations of cytokines and growth factors excreted into the receptor fluid after a single 4-hour dose with PFHxS or PFHxA (0.015%) (A-D). Bars represent mean (± SD) of 2-3 samples/group.

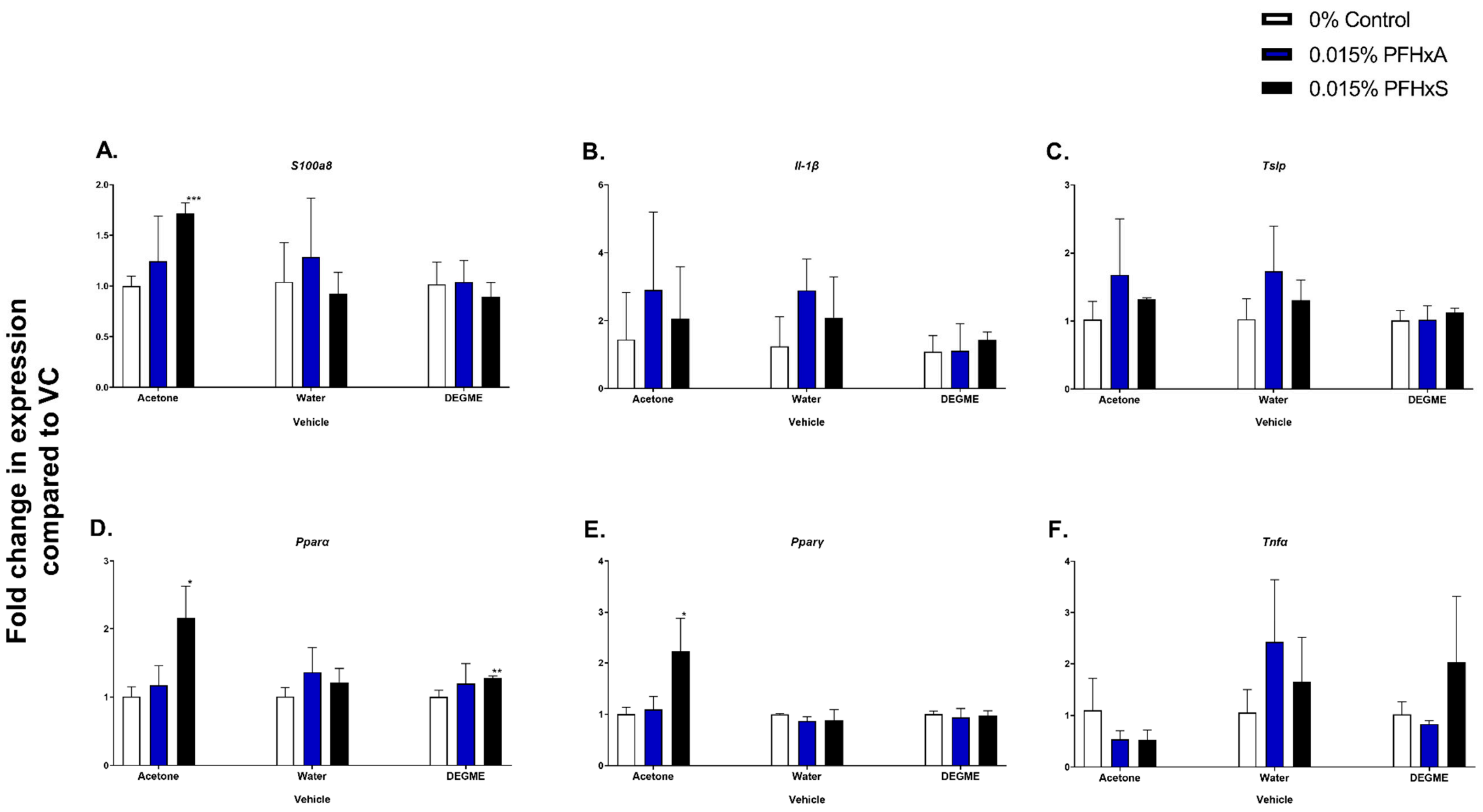

Supplemental Figure S4. Gene expression following a single 4-hour PFAS exposure to EpiDerm. Fold-change in gene expression compared to vehicle control of (A) *S100a8*, (B) *Il-1β*, (C) *Tslp*, (D) *Pparα*, (E) *Pparγ*, and (F) *Tnfα* following a single 4-hour exposure with PFHxS or PFHxA (0.015%) (A-F). Bars represent mean (± SD) of three samples/group, with statistical significance indicated as *$p < 0.05$, ***$p < 0.001$.

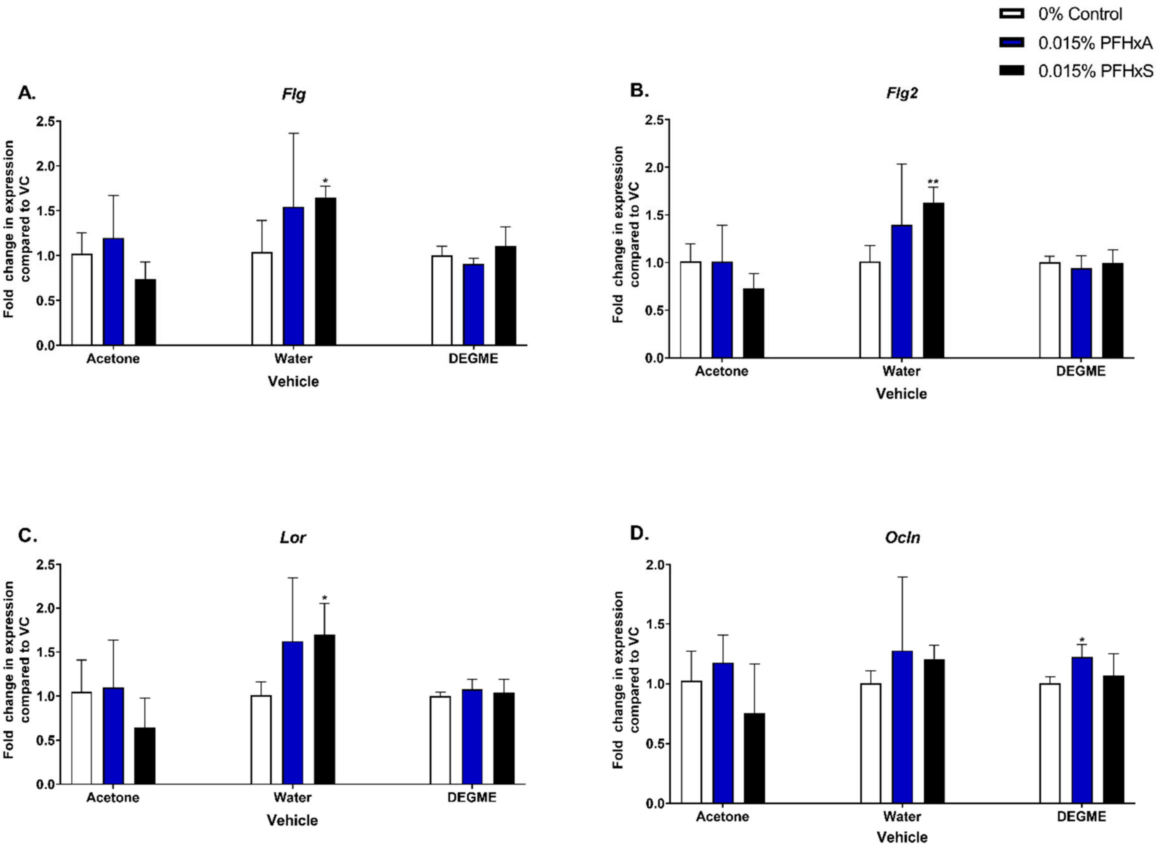

Supplemental Figure S5. Gene expression following a single 4-hour PFAS exposure to EpiDerm. Fold-change in gene expression compared to vehicle control of (A) *Flg*, (B) *Flg2*, (C) *Lor*, and (D) *Ocln* following a single 4-hour exposure with PFHxS or PFHxA (0.015%) (A-D). Bars represent mean (± SD) of 2-3 samples/group, with statistical significance indicated as *p < 0.05, **p < 0.01.
